# Melanoma exosomal miR-708-5p promotes macrophage M2 polarization and cancer metastasis

**DOI:** 10.1038/s41419-026-08597-1

**Published:** 2026-03-24

**Authors:** Meng Xu, Bincan He, Xiaofeng Zhou, Li Shu, Dan Ma

**Affiliations:** 1 Fudan University, Shanghai, China; 2 State Key Laboratory of Gene Expression, School of Life Sciences, Westlake University, Hangzhou, China; 3 Key Laboratory of Structural Biology of Zhejiang Province, School of Life Sciences, Westlake University, Hangzhou, China; 4 Westlake Laboratory of Life Sciences and Biomedicine, Hangzhou, China; 5 Institute of Biology, Westlake Institute for Advanced Study, Hangzhou, China

**Keywords:** Melanoma, Cancer microenvironment, Monocytes and macrophages, miRNAs

## Abstract

Monocyte-derived macrophages are usually recruited and play pivotal roles in establishing an immunosuppressive tumor microenvironment, and the interplay between tumor cells and tumor-associated macrophages (TAMs) is crucial for tumor development. However, the detailed mechanisms remain largely unelucidated in certain aggressive human cancers, such as melanoma. Here, through miRNA sequencing analysis, we found the microRNA miR-708-5p was highly enriched in melanoma exosomes, which was dependent on SFRS1. Treatment by melanoma exosomes facilitated M2 polarization of macrophages, while the polarized macrophages in turn promoted melanoma progression and metastasis both in vitro and in vivo. Mechanistically, miR-708-5p directly targets FOXN3, a member of the fork head/winged helix transcription factor family, and subsequently activates the PI3K/AKT/mTOR pathway in macrophages. Conversely, re-expression of FOXN3 in macrophages stably expressing miR-708-5p could reverse the impact on macrophages. In addition, downregulation of FOXN3 by miR-708-5p in macrophages reduced their phagocytic capacity and increased the secretion of IL-10 and TGF-β. Interestingly, we found that cellular retention of miR-708-5p could inhibit the proliferation and promote the apoptosis of melanoma cells, suggesting the necessity for secretion of this microRNA. In summary, our findings provide novel insights into the mechanism of melanoma-derived miR-708-5p in facilitating the formation of an immunosuppressive tumor microenvironment and indicate the potential of miR-708-5p and FOXN3 as therapeutic targets for the treatment of melanoma.

## Introduction

Melanoma is a highly malignant and aggressive type of skin cancer, which is characterized by its propensity for early metastasis. Lack of effective screening regimens often results in patients being diagnosed at an advanced metastatic stage [1]. The global incidence of melanoma is rising at an annual rate of 3–7%, and the 5-year survival rate for metastatic melanoma remains approximately 35%. Some immune checkpoint inhibitors, particularly PD-1 inhibitors, have improved clinical outcomes [2, 3], however, 30%–40% of patients either do not respond to these treatments or develop resistance, leading to a poor overall prognosis [4]. There is an urgent need for unraveling the fundamental mechanisms underlying melanoma metastasis and identifying novel biomarkers and therapeutic targets to facilitate the rapid and accurate diagnosis of melanoma at an early stage.

Bone marrow-derived monocytes are usually recruited to tumors including melanoma as tumor-associated macrophages (TAMs) [5]. TAMs can be classified into classically activated macrophages (M1-like macrophages) exhibiting phagocytic activity with tumor-killing effects, and alternatively activated macrophages (M2-like macrophages) that secrete immunosuppressive factors and facilitate tumor progression and metastasis [6–9]. Crosstalk between tumor cells and macrophages is extremely important for macrophages polarization, which can be mediated by exosomes. Tumor-derived exosomes play a crucial role in delivering different contents [10–12] to immune cells to modulate their function and thus establish a microenvironment conducive to tumor growth [13, 14]. One of the most important class of exosomal contents are microRNAs (miRNAs) [15, 16], which can intracellularly bind to target mRNAs bearing complementary sequences to downregulate the expression of corresponding genes [17]. Some miRNAs are known to induce M2-like macrophage polarization to promote cancer progression [18, 19], such as the following tumor-cell-originated miRNAs including miR-21-5p from hepatocellular carcinoma cells [20], miR-374b-3p derived from exosomes of glioblastoma stem cells [21], exosomal miR-138-5p from breast cancer cells [22] and miR-106a-5p from colorectal cancer cells [23]. Some other miRNAs may inhibit M2-type conversion of macrophages, for example, miR-199-5p and miR-204-5p have been found to inhibit tumor growth by directly reducing melanoma cell growth and indirectly hampering the recruitment and reprogramming of pro-tumoral macrophages [24]. However, how melanoma-cell-derived exosomes influence the function of macrophages and melanoma development, and whether it is dependent on specific miRNAs were not well elucidated.

Here, we report that melanoma-derived exosomes promote melanoma metastasis by facilitating the polarization of macrophages to the M2 phenotype, during which process miR-708-5p plays a crucial role. Initially identified in 2007, miR-708-5p is generated through the splicing of intron 1 of the ODZ4 gene [25], and has been suggested to be able to induce mood disorder-associated behavior in mice [26]. In this study, we found that miR-708-5p directly targets FOXN3 to activate the PI3K/AKT signaling pathway in macrophages to promote M2-polarization, highlighting another important regulatory function of this miRNA. The resulting M2-like macrophages release cytokines such as IL-10 and TGF-β, along with an upregulated expression of PD-L1, which collectively facilitate melanoma metastasis. These findings elucidate the molecular mechanisms by which melanoma-cell-derived exosomes influence macrophage polarization and contribute to melanoma development. Furthermore, miR-708-5p and its direct target FOXN3 might serve as potential biomarkers for early detection of melanoma or as therapeutic targets for melanoma treatment.

## Results

### Melanoma-cell-derived exosomes induce M2 polarization of macrophages

**Fig. 1:**
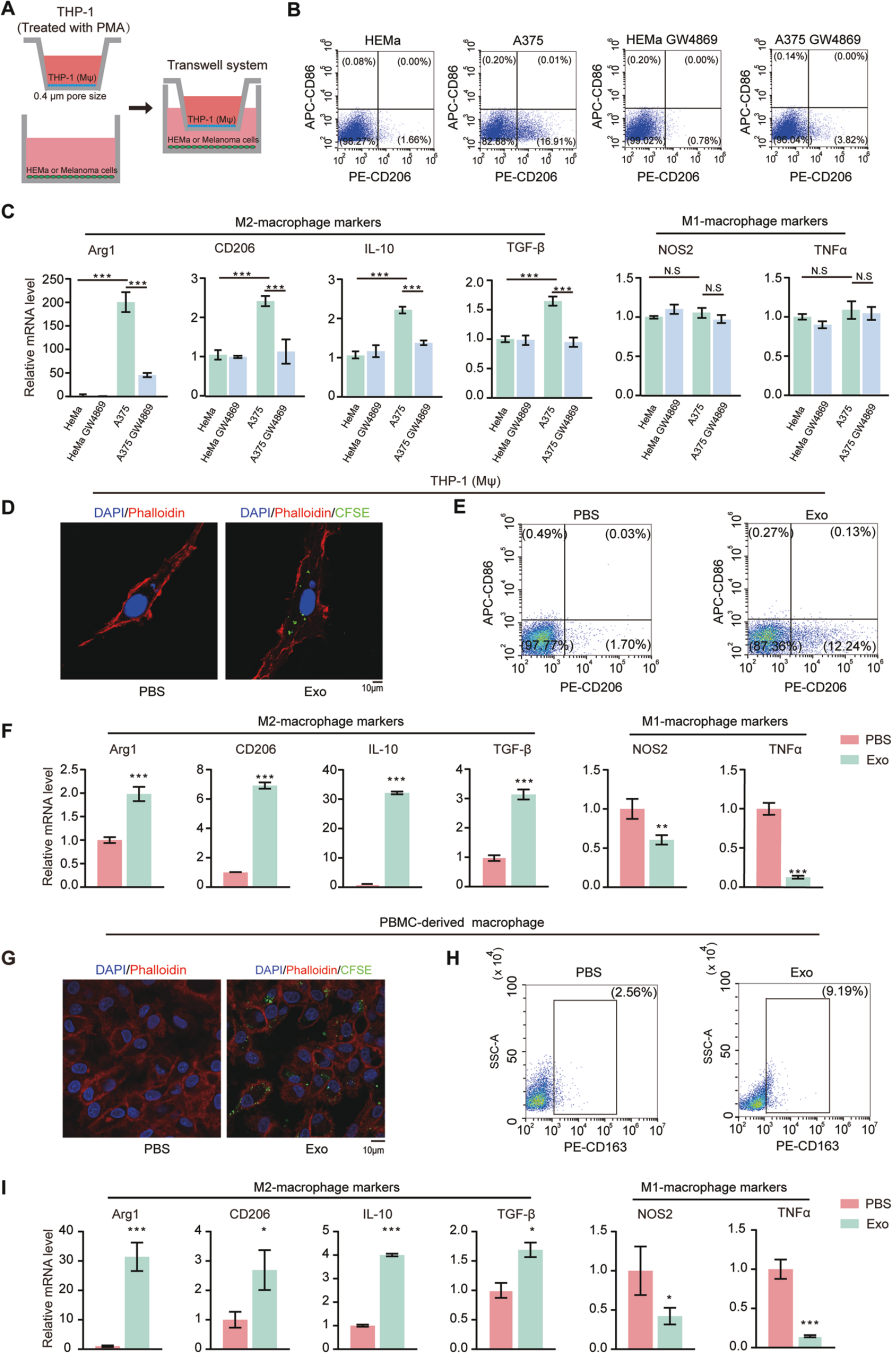
Melanoma-cell-derived exosomes induce M2 polarization of macrophages. **A** An illustration of the transwell system for co-culturing of HEMa or melanoma cells with macrophages using a transwell chamber with 0.4 μm pore size. HEMa or A375 cells were treated with or without 5 μM of exosome secretion inhibitor GW4869. THP-1 (Mφ) macrophages induced with PMA (50 ng/ml) from THP-1 cells in the upper chamber, were co-cultured with HEMa or A375 melanoma cells in the lower chamber for 48 h. Only conditioned media could be exchanged between the two chambers. **B** Representative dot plots from flow cytometry analysis of the proportion of CD206^+^ macrophages in THP-1 (Mφ) when co-cultured with HEMa or A375 cells, with or without GW4869 treatment. **C** Analysis using qRT-PCR of M2 markers (Arg1, CD206, IL-10, TGF-β) and M1 markers (NOS2, TNF-α) in THP-1 (Mφ) under different co-culturing conditions. Data are presented as mean ± SD (*n* = 3), and statistical significance was assessed by a one-way ANOVA with Tukey’s multiple comparisons test. *P < 0.05; ***P < 0.001. N.S, not significant. **D** Representative immunofluorescence staining of phalloidin labeled cytoskeleton (red) and CFSE-labeled exosomes (green) for THP-1 (Mφ) with or without exosome treatment. Scale bar, 10 μm. **E** Representative dot plots of flow cytometry analysis of macrophages stained with PE-CD206 and APC-CD86. THP-1 (Mφ) were treated with isolated A375-derived exosomes or PBS for 24 h. Result for PBS-treated cells was used as a negative control. **F** Analysis using qRT**-**PCR of M2 markers (Arg1, CD206, IL-10, TGF-β) and M1 markers (NOS2, TNF-α) in THP-1 (Mφ) treated with A375-derived exosomes or PBS. Data are presented as mean ± SD (*n* = 3), and statistical significance was assessed by an unpaired student’s *t* test. *P < 0.05; ***P < 0.001. **G** Representative immunofluorescence staining of phalloidin-labeled cytoskeleton (red) and CFSE-labeled exosomes (green) for PBMC-derived macrophages with or without exosome treatment. Scale bar, 10 μm. **H** Representative dot plots of flow cytometry analysis of macrophages stained with PE-CD163(M2-marker). PBMC-derived macrophages were treated with isolated A375 exosomes or PBS for 24 h. Result for PBS-treated cells was used as a negative control. **I** Analysis using qRT-PCR of M2 markers (Arg1, CD206, IL-10, TGF-β) and M1 markers (NOS2, TNF-α) in PBMC-derived macrophages treated with A375-derived exosomes or PBS. Data are presented as mean ± SD (*n* = 3), and statistical significance was assessed by an unpaired student’s *t* test. *P < 0.05; ***P < 0.001.

To investigate the crosstalk between melanoma cells and macrophages, we co-cultured the human melanoma cell line A375 or the control cell line of human epidermal melanocytes (HEMa) with the PMA-induced human THP-1 cells (THP-1 (Mφ)) in a transwell system (Fig. 1A). Flow cytometry analysis revealed that co-culture with A375 cells increased the percentage of THP-1 (Mφ) cells expressing the M2-macrophage marker CD206 (CD206^+^) (Fig. 1B). In contrast, when treated with GW4869, a widely used exosome release inhibitor, the expression of CD206 was significantly reduced (Fig. 1B). qRT-PCR analysis further demonstrated that, the expression of M2-macrophage markers (Arg1, CD206, IL-10 and TGF-β) was significantly elevated in THP-1(Mφ) cells co-cultured with A375, while the expression of M1-macrophage markers (NOS2 and TNF-α) remained unchanged (Fig. 1C). Conversely, when treated with GW4869, the expression of M2-macrophage markers was significantly decreased (Fig. 1C). These findings suggest that melanoma cells induce M2 polarization of macrophages, likely through the release of exosomes.

To investigate whether melanoma-derived exosomes influence macrophage polarization, we first isolated exosomes from A375 cells through a centrifugation-based method (Supplementary Fig. 1A). Western blot results showed that exosomal markers (CD9, CD63, TSG101, and Alix) were enriched, whereas the Golgi matrix protein GM130 was undetectable (Supplementary Fig. 1B). The products exhibited classic- cup shaped structure (Supplementary Fig. 1C) with an average diameter of 130 nm (Supplementary Fig. 1D), consistent with previous studies [27], indicating successful isolation of exosomes with high purity. We treated phalloidin-labeled THP-1(Mφ) with CFSE-labeled exosomes to check exosome engulfment by confocal microscopy and observed exosome internalization by the macrophages (Fig. 1D). Then, the influences of the A375-derived exosomes on THP-1 (Mφ) polarization were examined. Flow cytometric analysis demonstrated that the proportion of CD206^+^ THP-1 (Mφ) was significantly higher upon exosome treatment, while the expression level of the M1 maker CD86 was similar compared to the control (Fig. 1E). Additionally, qRT-PCR analysis revealed that the mRNA levels of M2-markers including Arg1, CD206, IL-10, and TGF-β were significantly increased, whereas that of M1-markers of NOS2 and TNF-α were dramatically decreased upon exosomes treatment (Fig. 1F). Similar results were also obtained in peripheral-blood-mononuclear-cell-derived (PBMC-derived) macrophages (Fig. 1G–I and Supplementary Fig. 1E) and RAW264.7 cells (Supplementary Fig. 1F–H) treated by A375- and B16F10-derived exosomes respectively. These results suggested that melanoma-derived exosomes could effectively promote M2 polarization of macrophages.

### Melanoma exosomal miR-708-5p mediates M2 polarization of macrophages

**Fig. 2:**
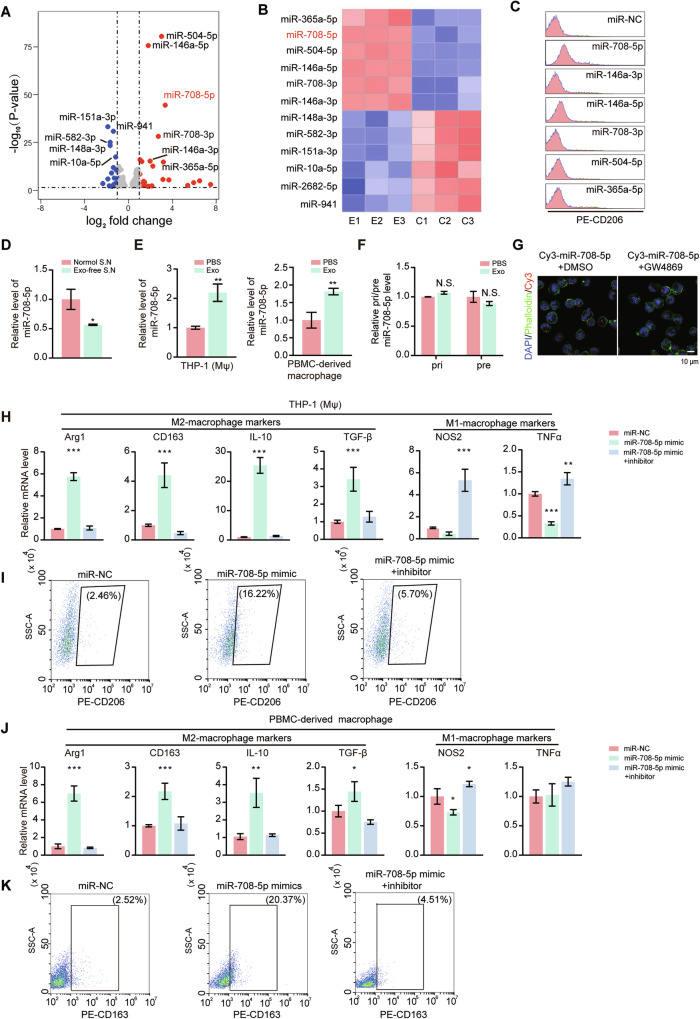
Melanoma exosome enriched miR-708-5p is internalized by macrophages and promotes their M2 polarization. **A** The volcano map of miRNA-seq showing differentially expressed miRNAs in THP-1 (Mφ) treated with or without A375-derived exosomes. **B** Heatmap of the differentially expressed miRNAs in (**A**). **C** Flow cytometry analysis of the proportion of CD206^+^ macrophages in THP-1 (Mφ) transfected with mimics of selected miRNAs that were upregulated. **D** Relative level of miR-708-5p in THP-1 (Mφ) treated with the supernatant (S.N.) of A375 cells with or without exosome depletion by ultracentrifugation. **E** Relative miR-708-5p level within THP-1 (Mφ) and PBMC-derived macrophages after treatment with PBS or A375-derived exosomes. **F** Relative pri-miR-708-5p and pre-miR-708-5p levels within THP-1 (Mφ) after treatment with PBS or A375-derived exosomes. **G** Representative immunofluorescence staining of phalloidin-labeled cytoskeleton (green) and Cy3-miR-708-5p (red) with THP-1 (Mφ) macrophages after 48 h co-culture with A375 cells treated with DMSO or the exosome secretion inhibitor GW4869. Scale bar, 10 μm. **H** Relative mRNA level of M2 markers (Arg1, CD163, IL-10, TGF-β) and M1 markers (NOS2, TNF-α) in THP-1 (Mφ) transfected with miR-708-5p mimic, miR-708-5p inhibitor or a miRNA negative control (miR-NC). **I** Proportion of CD206^+^ macrophages in THP-1 (Mφ) transfected with miR-NC, miR-708-5p mimic or miR-708-5p inhibitor. **J** Relative mRNA level of M2 markers (Arg1, CD163, IL-10, TGF-β) and M1 markers (NOS2, TNF-α) in PBMC-derived macrophages transfected with miR-708-5p mimic, miR-708-5p inhibitor or a miRNA negative control (miR-NC). **K** Proportion of CD163^+^ macrophages in PBMC-derived macrophages transfected with miR-NC, miR-708-5p mimic or co-transfected with miR-708-5p mimic and miR-708-5p inhibitor.

To investigate whether miRNAs carried by melanoma exosomes are involved in promoting M2 polarization of macrophages, we performed miRNA sequencing of THP-1 (Mφ) cells after exosome treatment. 25 miRNAs were found to be upregulated and 19 were downregulated ( | log_2_FC | > 1, *P* Value < 0.05) in THP-1 (Mφ) treated with A375-derived exosomes. The top six significantly upregulated or downregulated miRNAs were displayed in volcano plot and heatmap (Fig. 2A, B). We stably expressed the top six upregulated miRNA in THP-1 (Mφ), and flow cytometry analysis confirmed that M2 macrophage marker CD206 was significantly upregulated when miR-708-5p was overexpressed (Fig. 2C).

To explore whether miR-708-5p upregulation in THP-1 (Mφ) directly results from the uptake of melanoma-derived exosomes, we assessed miR-708-5p level under distinct conditions. We compared the relative levels of intracellular and exosomal miR-708-5p of A375 and B16F10 cells, and found that miR-708-5p was significantly enriched in exosomes released by both cells (Supplementary Fig. 2A). qRT-PCR analysis confirmed the enrichment of miR-708-5p in exosome-containing A375 conditioned media but its level was decreased in exosome depleted media by ultracentrifugation (Fig. 2D). Additionally, there was a significant increase of miR-708-5p level in THP-1 (Mφ), PBMC-derived macrophages or Raw264.7 after the treatment with A375 or B16F10-derived exosomes (Fig. 2E and Supplementary Fig. 2B). To further confirm the origin of the enriched miR-708-5p in the exosome-treated macrophages, we assessed the levels of the precursors of miR-708-5p including pri-miRNA and pre-miRNA in THP-1 (Mφ) with or without exosome treatment and no significant changes were observed (Fig. 2F), suggesting that the miR-708-5p enriched in the macrophages was from melanoma-derived exosomes but not endogenous expression. We then co-cultured THP-1 (Mφ) cells with A375 cells transfected with Cy3-labeled miR-708-5p, with or without GW4869 treatment, and confocal microscopy analysis suggested that the internalization of Cy3-labeled miR-708-5p by THP-1 (Mφ) was obviously decreased with GW4869 treatment (Fig. 2G). Above results suggested that miR-708-5p were enriched in melanoma exosomes and further internalized by macrophages.

To further investigate the impact of miR-708-5p on macrophage polarization, we transfected miR-708-5p mimic or inhibitor into THP-1 (Mφ) cells. qRT-PCR analysis revealed that, overexpression of miR-708-5p significantly increased the expression of M2 markers while decreased the expression of M1 markers (Fig. 2H), with the mRNA level of M2 markers markedly decreased when miR-708-5p inhibitor was co-transfected (Fig. 2H). Flow cytometry analysis further revealed that miR-708-5p significantly upregulated CD206 level in macrophages, while this effect was inhibited by the miR-708-5p inhibitor (Fig. 2I). Similar results were also obtained from PBMC-derived macrophages (Fig. 2J, K) and Raw264.7 cells (Supplementary Fig. 2C, D). These findings suggest that melanoma-cell-derived miR-708-5p facilitates the polarization of macrophages towards the M2 phenotype.

### miR-708-5p function as a tumor suppressor in melanoma

To investigate the direct influences of miR-708-5p in melanoma cells, we compared its expression levels across several melanoma cell lines. Interestingly, we found that miR-708-5p level was decreased within SK-MEL-2, SK-MEL-28, and A375 cells while significantly elevated in corresponding exosomes (Supplementary Fig. 3A), suggesting that the enrichment of miR-708-5p in exosomes is a common phenomenon among melanoma cell lines. Next, we transfected A375, SK-MEL-2 and SK-MEL-28 cells with either control miRNA or miR-708-5p mimic. Western blot analysis demonstrated that the protein level of Cyclin B, a key regulatory protein controlling cell cycle progression, was significantly decreased in tumor cells overexpressing miR-708-5p, indicating that miR-708-5p suppress tumor-cell cycle (Supplementary Fig. 3B). Additionally, we analyzed the apoptosis levels of the melanoma cells and found that miR-708-5p overexpression in A375 cells significantly increased the proportion with elevated expression of the early apoptosis marker annexin V and the late apoptosis marker 7-AAD, which effects could be reversed by the miR-708-5p inhibitor (Supplementary Fig. 3C). Wound healing assays further revealed that miR-708-5p suppresses A375 cell migration (Supplementary Fig. 3D). Furthermore, A375 cells with miR-708-5p overexpression exhibited decreased cell proliferation (Supplementary Fig. 3E). To confirm that miR-708-5p exerts similar effects in vivo, we subcutaneously injected nude mice with A375 cells transfected with miR-NC or miR-708-5p mimic and measured tumor volume every two days. The results showed that miR-708-5p overexpression in the melanoma cells significantly reduced tumor growth in vivo (Supplementary Fig. 3F). These findings suggest that miR-708-5p may function as a tumor suppressor and inhibit melanoma growth, and the secretion of miR-708-5p through exosomes may also play a crucial role in relieving such inhibitory effects.

### miR-708-5p was packaged in exosomes by SFRS1

**Fig. 3:**
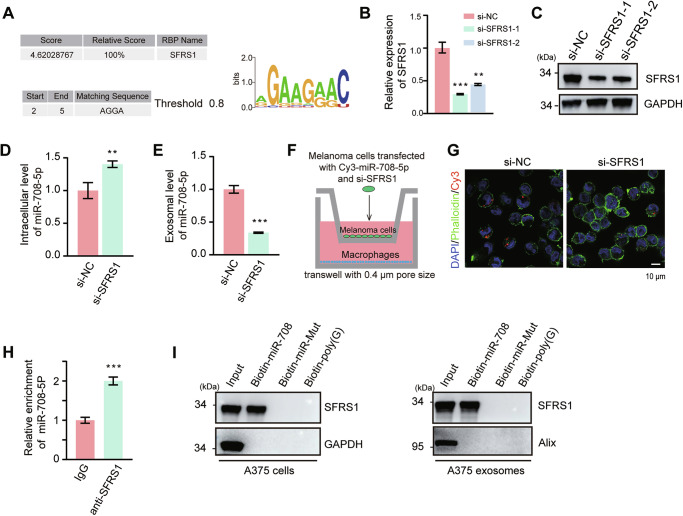
miR-708-5p was packaged into exosomes by SFRS1. **A** Prediction of miR-708-5p binding protein and the potential matching sequence in miR-708-5p with RBPDB (left) and RBPsuite (right). **B** qRT-PCR examination of siRNA-mediated knockdown efficiency of SFRS1 at mRNA level. **C** Western blot examination of siRNA-mediated knockdown efficiency of SFRS1 at protein level. **D** The intracellular level of miR-708-5p in A375 cells transfected with si-NC or si-SFRS1. **E** The miR-708-5p level in A375-derived exosomes when A375 cells were transfected with si-NC or si-SFRS1. **F** THP-1 (Mφ) were co-cultured with A375 cells pre-transfected with si-NC or si-SFRS1 and Cy3-miR-708-5p (red). **G** Immunofluorescence staining was performed to detect the red fluorescent signals in THP-1 (Mφ). Scale bar, 10 μm. **H** Relative enrichment of miR-708-5p by IgG (negative control) or SFRS1-immobilized resin through RIP assay. **I** Western blot examination of SFRS1 pull-down from A375 cell lysates or exosomes by biotinylated miR-708-5p, miR-708-5p mutant or polyG. In (**B**), data are presented as mean ± SD (*n* = 3), and statistical significance was assessed by a one-way ANOVA with Tukey’s multiple comparisons test. **P < 0.01; ***P < 0.001. In (**D**, **E**, **H**), data are presented as mean ± SD (*n* = 3), and statistical significance was assessed by an unpaired student’s *t* test. *P < 0.05; **P < 0.01; ***P < 0.001.

To investigate the secretion mechanism of miR-708-5p, we used RBPDB and RBPsuite servers to predict the binding proteins of miR-708-5p and identified SFRS1 as a potential candidate involved in the exosomal sorting of miR-708-5p (Fig. 3A). We knocked down SFRS1 in A375 cells (Fig. 3B, C) and analyzed the effects on the localization of miR-708-5p. qRT-PCR results indicated that in SFRS1-knockdown cells, miR-708-5p accumulates in cells (Fig. 3D) rather than being sorted into exosomes (Fig. 3E). Thereafter, we transfected Cy3-labeled miR-708-5p into wild-type or SFRS1-knockdown A375 cells and co-cultured with THP-1 (Mφ) cells through the transwell system. Immunofluorescence analysis demonstrated a significant decrease in the internalization of Cy3-labeled miR-708-5p by THP-1 (Mφ) cells following SFRS1 knockdown (Fig. 3F, G). To further substantiate this finding, A375 cell lysates were incubated with either IgG or SFRS1-preconjugated resin for RNA immunoprecipitation (RIP). qRT-PCR analysis confirmed the interaction between SFRS1 and miR-708-5p (Fig. 3H). Additionally, miRNA pull-down experiments suggested that miR-708-5p binding with SFRS1, while the binding was eliminated upon mutation of the predicted binding sequence (AGGA) in miR-708-5p (Fig. 3I). These results suggest that SFRS1 binds to miR-708-5p and mediates its sorting into melanoma exosomes for secretion.

### miR-708-5p delivered into macrophages promote melanoma progression and metastasis

**Fig. 4:**
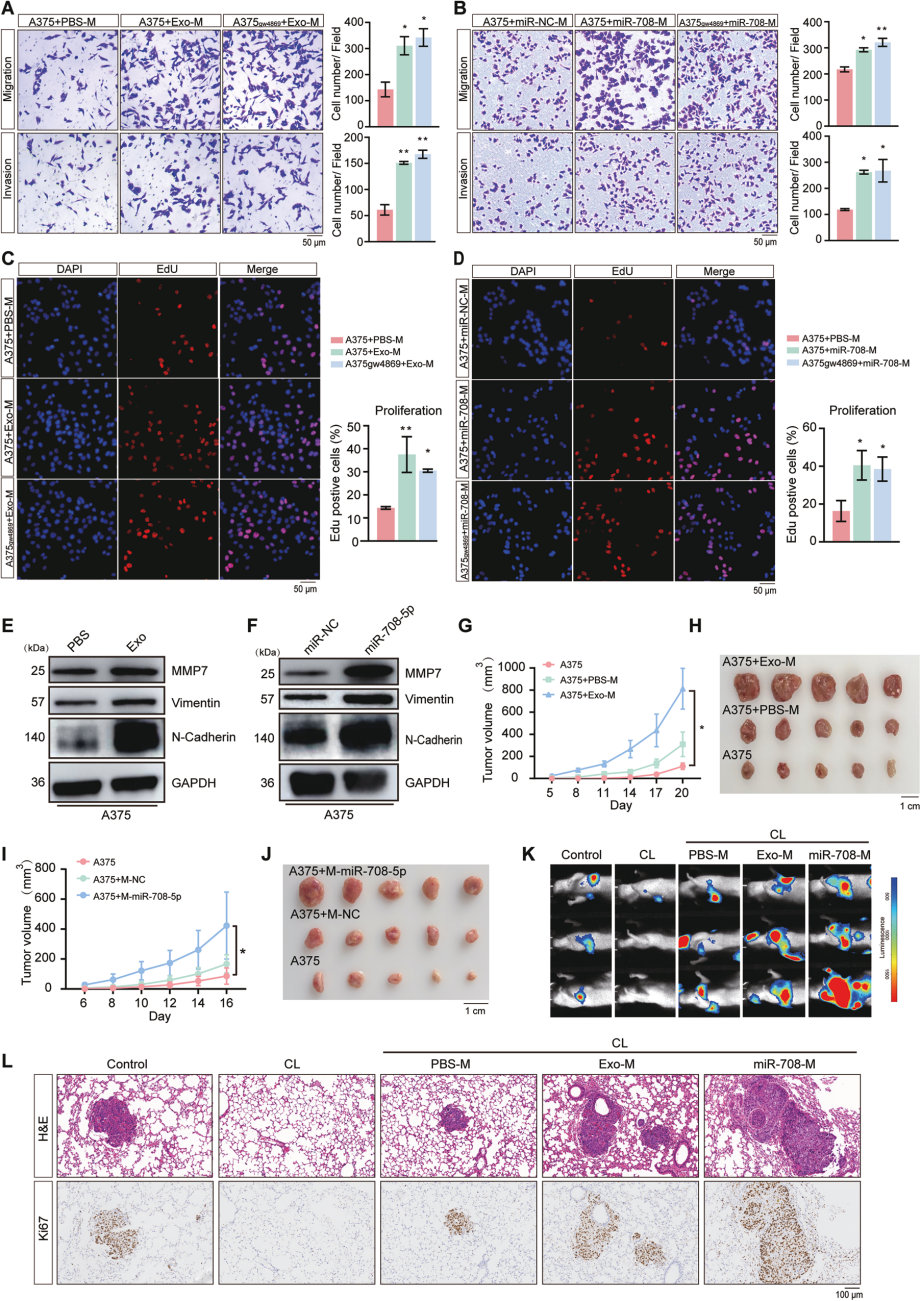
Treatment with melanoma exosomes or miR-708-5p expression in macrophages promote melanoma progression and metastasis. **A** Invasion and migration capabilities of A375 cells (with or without GW4869 treatment) co-cultured with conditioned macrophages (PBS-M, THP-1 (Mφ) pre-treated with PBS; Exo-M, THP-1 (Mφ) pre-treated with A375-derived exosomes) were determined by the transwell co-culture system. Scale bar, 50 μm. Cell numbers are presented as mean ± SD, and statistical significance was assessed by a one-way ANOVA tes*t*. ***P < 0.001. **B** Invasion and migration capabilities of A375 cells (with or without GW4869 treatment) co-cultured with conditioned macrophages (miR-NC-M, THP-1 (Mφ) transfected with miR-NC; miR-708-M, THP-1 (Mφ) transfected with miR-708-5p mimic) was determined by the transwell co-culture system. Scale bar, 50 μm. Cell numbers are presented as mean ± SD, and statistical significance was assessed by a one-way ANOVA tes*t*. ***P < 0.001. **C** Proliferation profiles of A375 cells (with or without GW4869 treatment) that were incubated with the supernatants of macrophages pre-treated with PBS or exosomes. Scale bar, 100 μm. Percentage of EdU-positive cells are presented as mean ± SD, and statistical significance was assessed by a one-way ANOVA tes*t*. **P < 0.01. **D** Proliferation profiles of A375 cells (with or without GW4869 treatment) incubated with the supernatants of macrophages transfected with miR-NC or miR-708-5p mimic. Scale bar, 100 μm. Percentage of EdU-positive cells are presented as mean ± SD, and statistical significance was assessed by a one-way ANOVA tes*t*. **P < 0.01. **E** The effects of the supernatants of THP-1 (M**φ**) pretreated with PBS or exosomes on the EMT of A375 cells were analyzed by Western blot. **F** The effects of the supernatants of THP-1 (Mφ) transfected with miR-NC or miR-708-5p mimic on the EMT of A375 cells were analyzed by Western blot. **G**, **H** Tumors from xenograft nude mice implanted with A375, A375 with PBS treated-macrophages or exosome-treated macrophages were recorded every three days (*n* = 5), and tumor were isolated for comparison. Statistical significance was determined using one-way ANOVA test. *P < 0.05. **I**, **J** Tumors from xenograft nude mice implanted with A375, A375 with macrophages transfected with miR-NC or miR-708-5p were recorded every two days (*n* = 5), and tumor were isolated for comparison. Statistical significance was determined using one-way ANOVA test. *P < 0.05. **K** In vivo bioluminescent imaging analysis of lung metastasis in nude mice pre-treated with CL and injected with A375-luciferase, A375-luciferase with PBS treated macrophages, A375-luciferase with A375 exosomes or miR-708-5p-treated macrophages via tail vein injection. Representative images are shown (*n* = 3). **L** Images of IHC staining for Ki67 and H&E staining of neighboring sections in lung tissues from the indicated mice in (**K**). Scale bar, 100 μm.

TAMs are recognized for their ability to promote melanoma progression [28]. We used the transwell co-culture system (Supplementary Fig. 4A) to determine whether polarized macrophages by exosomal miR-708-5p influence the migration and invasion capabilities of melanoma cells in vitro. THP-1 (Mφ) or Raw264.7 cells pre-treated with exosomes or transfected with miR-708-5p mimic, were co-cultured with A375 or B16F10 cells respectively. GW4869 was used to inhibit the secretion of exosomes, thereby eliminating the potential effects generated through autocrine signaling by newly generated melanoma exosomes. The results suggested that melanoma cells were more capable in invasion and migration (Fig. 4A, B and Supplementary Fig. 4B) and exhibited higher proliferation rate (Fig. 4C, D and Supplementary Fig. 4C) with exosome incubation or miR-708-5p transfection, regardless of GW4869 treatment. In addition, we treated A375 cells with the supernatant of macrophages that were pre-treated with melanoma-derived exosomes or expressed the miR-708-5p, and the expression level of EMT-associated proteins were found to be upregulated in A375 cells (Fig. 4E, F). These results indicate that polarized macrophages by treatment with melanoma-derived exosomes or overexpressing miR-708-5p promote the migration, invasion, and proliferation of melanoma cells in vitro.

We also examined the influence of THP-1 (Mφ) macrophages polarized by A375-exosome treatment or miR-708-5p overexpression on melanoma growth in vivo, through co-injection of corresponding macrophages with A375 cells into nude mice. The tumor growth rates and sizes in the exosome-treated or miR-708-5p-expressing groups were significantly greater than those in the corresponding control groups (Fig. 4G–J). These results demonstrated that polarized macrophages by miR-708-5p promote the proliferation of melanoma in vivo. We further depleted endogenous macrophages in nude mice with clodronate liposomes (CL), and monitor the effects of THP-1 (Mφ) macrophages polarized by A375-exosome treatment or miR-708-5p overexpression on tumor metastasis through co-injection with A375-luciferase cells into nude mice. Bioluminescent imaging revealed that co-injection of A375 cells with THP-1 (Mφ) cells pre-treated with exosomes or overexpressed miR-708-5p, markedly accelerated lung metastasis in mice (Fig. 4K). Immunohistochemical staining for Ki67 and H&E staining showed a significant increase in both the size and number of lung metastases (Fig. 4L). These findings suggest that the M2 polarization of macrophages, mediated by miR-708-5p, in turn promotes melanoma development.

### miR-708-5p directly targets FOXN3 in macrophages

**Fig. 5:**
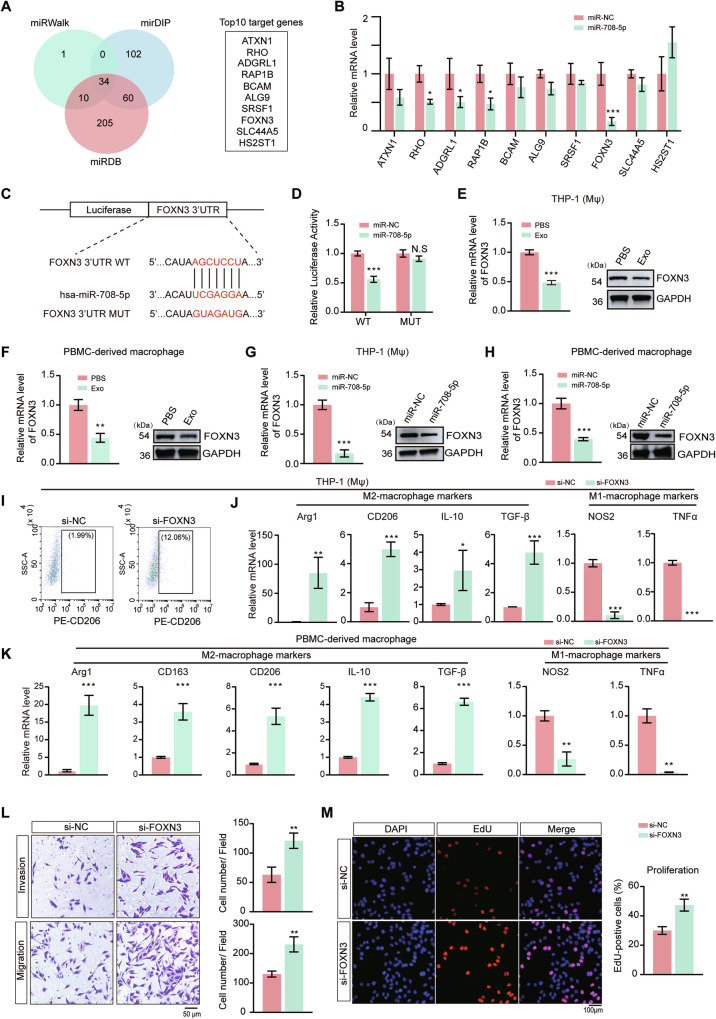
Identification of FOXN3 as the direct target of miR-708-5p and the roles of FOXN3. **A** Venn diagram showing the predicted miR-708-5p target genes in three databases including miRWalk, mirDIP and miRDB. TOP10 target genes in the three databases are listed. **B** Analysis using qRT-PCR of the indicated genes in THP-1 (Mφ) transfected with miR-NC or miR-708-5p mimic. Data are presented as mean ± SD (*n* = 3), and statistical significance was assessed by an unpaired student’s t test. *P < 0.05; ***P < 0.001. **C** Schematic diagram of the binding between miR-708-5p and the FOXN3 3’-UTR and the design of a luciferase reporting system to confirm the interaction. **D** Relative luciferase activity of cells bearing WT or mutant (MUT) FOXN3 luciferase reporters when miR-NC or miR-708-5p mimic were transfected. **E**–**F** Relative mRNA level and western blot examination of FOXN3 in THP-1 (Mφ) and PBMC-derived macrophages treated with PBS or A375-derived exosomes. Data are presented as mean ± SD (*n* = 3), and statistical significance was assessed by an unpaired student’s *t* test. ***P < 0.001. **G**, **H** Relative mRNA level and western blot examination of FOXN3 in THP-1 (Mφ) and PBMC-derived macrophages transfected with miR-NC or miR-708-5p mimic. Data are presented as mean ± SD (*n* = 3), and statistical significance was assessed by an unpaired student’s *t* test. ***P < 0.001. **I** Representative dot plots from flow cytometry analysis of the proportion of CD206^+^ macrophages in THP-1 (Mφ) transfected with si-NC or si-FOXN3. **J**, **K** Relative mRNA level of M2 markers (Arg1, CD206, IL-10, TGF-β) and M1 markers (NOS2, TNFα) in THP-1 (Mφ) and PBMC-derived macrophages transfected with si-NC or si-FOXN3. Data are presented as mean ± SD (*n* = 3), and statistical significance was assessed by an unpaired student’s *t* test. *P < 0.05; **P < 0.01; ***P < 0.001. **L** Invasion and migration capabilities of A375 cells co-cultured with conditioned macrophages transfected with si-NC or si-FOXN3. Scale bar, 50 μm. Data are presented as mean ± SD, and statistical significance was assessed by an unpaired student’s *t* test. **P < 0.01. **M** The proliferation of A375 cells treated with the supernatants of THP-1 (Mφ) transfected with si-NC or si-FOXN3. EdU was used to stain active proliferating cells. Scale bar, 100 μm. Data are presented as mean ± SD, and statistical significance was assessed by an unpaired student’s *t* test. **P < 0.01.

To identify the direct targets of miR-708-5p in macrophages, three bioinformatics tools for miRNA target gene prediction were used and the results were shown in a Venn diagram (Fig. 5A), which revealed 34 overlapped potential target genes. We examined the mRNA expression levels of the top ten predicted genes and found that when miR-708-5p mimic was transfected into THP-1 (Mφ), the mRNA level of RHO, ADGRL1, RAP1B and FOXN3 were decreased, among which FOXN3 was the most significantly downregulated gene (Fig. 5B). It is well established that miRNAs downregulate the expression of target genes by binding to complementary sequences in the 3’-UTRs of corresponding mRNA transcripts [29]. Sequence analysis revealed a putative binding site for miR-708-5p within the 3’-UTR of FOXN3 (Fig. 5C). To validate the interaction, we generated a luciferase reporter system with wild-type FOXN3 3’-UTR or its mutant carrying mutations in the predicted miR-708-5p binding site, and cloned to the downstream of the luciferase gene (Fig. 5C). We found that miR-708-5p significantly reduced luciferase activity in cells transfected with the reporter containing wild-type FOXN3 3’-UTR, whereas no obvious change was observed when the reporter carrying FOXN3 3’-UTR mutant was transfected, indicating specific targeting of FOXN3 by miR-708-5p (Fig. 5D). Similar results were also observed in Raw264.7 cells treated with exosomes derived from B16F10 cells (Supplementary Fig. 5A). We also examined whether the rest three downregulated genes directly bind to miR-708-5p using similar luciferase reporter systems, and the results suggested no obvious binding between miR-708-5p and the 3’-UTR regions of RHO, ADGRL1 or RAP1B (Supplementary Fig. 5B).

Additionally, both qRT-PCR and western blot analysis demonstrated that FOXN3 expression was reduced in THP-1 (Mφ), PBMC-derived macrophages and RAW264.7 cells following the treatment with exosomes or expression of miR-708-5p mimic (Fig. 5E–H and Supplementary Fig. 5C), whereas the FOXN3 downregulation due to exosome treatment could be rescued by the miR-708-5p inhibitor (Supplementary Fig. 5D, E). Above findings suggest that miR-708-5p directly target FOXN3 in macrophages.

To further understand the relationship between FOXN3 and macrophage polarization, we knocked down the FOXN3 expression with small interfering RNAs (siRNAs) in macrophages and analyzed the knockdown efficiency by western blotting (Supplementary Fig. 5F). Downregulation of FOXN3 in THP-1 (Mφ), PBMC-derived macrophages or Raw264.7, significantly increased the M2 markers while decreased the M1 makers in corresponding macrophages (Fig. 5I–K and Supplementary Fig. 5G, H), suggesting that the downregulation of FOXN3 promotes macrophage polarization to the M2 phenotype. Subsequently, we transfected si-NC or si-FOXN3 into THP-1 (Mφ) and co-cultured with A375 to verified whether the knockdown of FOXN3 in macrophages promote melanoma progression. As expected, loss function of FOXN3 in macrophages promote A375 migration, invasion and proliferation (Fig. 5L, M). Immune infiltration analysis also revealed that the expression level of FOXN3 was negatively correlated with the infiltration of M2 macrophages but positively correlated with CD8^+^ T cells (Supplementary Fig. 5I). M2 macrophages typically express PD-L1 to mediate immunosuppression and play critical roles in tumor immune evasion, autoimmune regulation, and chronic inflammation [30], we hypothesized that FOXN3 deficiency may regulate PD-L1 expression in macrophages. Flow cytometry analysis showed that knocking down FOXN3 increased PD-L1 expression in macrophages which supported our hypothesis (Supplementary Fig. 5J). Furthermore, after co-culturing apoptotic A375 cells and THP-1 (Mφ) cells with or without FOXN3 knockdown, we observed that FOXN3 deficiency suppressed the phagocytic activity of macrophages (Supplementary Fig. 5K). These findings suggest that FOXN3 plays a critical role in regulating macrophage polarization and function, and its downregulation by miR-708-5p promotes a pro-tumor M2 phenotype of macrophages, thereby facilitating melanoma progression.

### FOXN3 deficiency promotes M2 polarization of macrophages by activating the PI3K/AKT signaling pathway

**Fig. 6:**
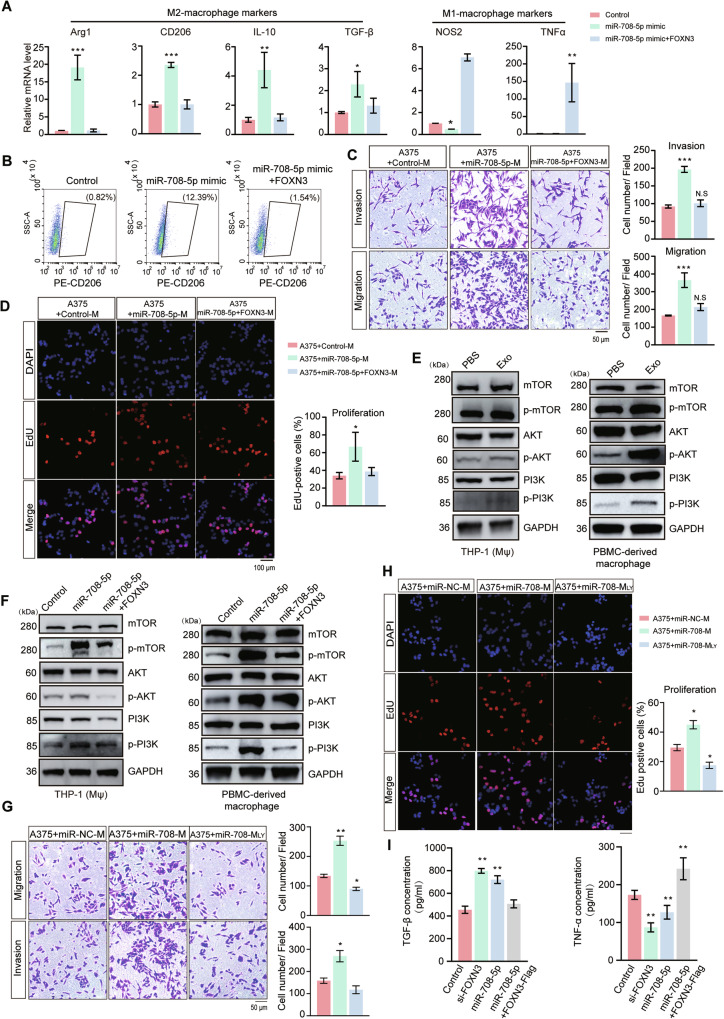
FOXN3 deficiency promotes macrophage polarization to the M2 phenotype by upregulating the PI3K/AKT signaling pathway. **A** Relative mRNA level of M2 markers (Arg1, CD206, IL-10, TGF-β) and M1 markers (NOS2, TNF-α) in THP-1 (Mφ) transfected with NC, miR-708-5p or miR-708-5p following with flag-FOXN3. Data are presented as mean ± SD (*n* = 3), and statistical significance was assessed by a one-way ANOVA with Tukey’s multiple comparisons test. *P < 0.05; **P < 0.01; ***P < 0.001. **B** Representative dot plots from flow cytometry analysis of the proportion of CD206^+^ macrophages in THP-1 (Mφ) treated in the same way as described in (**A**). **C** Invasion and migration capabilities of A375 cells co-cultured with THP-1 (Mφ) transfected with NC, miR-708-5p or miR-708-5p following with flag-FOXN3. Scale bar, 50 μm. Data are presented as mean ± SD, and statistical significance was assessed by a one-way ANOVA with Tukey's multiple comparisons tes*t*. ***P < 0.001. **D** The proliferation of A375 cells treated with the supernatants of THP-1 (Mφ) transfected with NC, miR-708-5p or miR-708-5p following with flag-FOXN3 were evaluated by EdU assay. Scale bar, 100 μm. Data are presented as mean ± SD, and statistical significance was assessed by a one-way ANOVA with Tukey's multiple comparisons tes*t*. *P < 0.05. **E** Western blot examination of the level of related proteins in PI3K/AKT/mTOR pathway in THP-1 (Mφ) and PBMC-derived macrophages treated with PBS or A375 exosomes. **F** The level of related proteins in PI3K/AKT/mTOR pathway in THP-1 (Mφ) and PBMC-derived macrophages transfected with NC, miR-708-5p or miR-708-5p following with flag-FOXN3. **G** Invasion and migration capabilities of A375 cells co**-**cultured with THP-1 (Mφ) cells transfected with miR-NC, miR-708-5p mimic, or miR-708-5p mimic and LY294002. Scale bar, 50 μm. **H** The proliferation of A375 cells treated with the supernatants of THP-1 (Mφ) transfected with miR-NC, miR-708-5p mimic, or miR-708-5p mimic and LY294002 were evaluated by EdU assay. Scale bar, 100 μm. Statistical data in panels (**G**) and (**H**) are presented as mean ± SD, and statistical significance was assessed by a one-way ANOVA with Tukey's multiple comparisons tes*t*. *P < 0.05; **P < 0.01; ***P < 0.001. **I** ELISA examination of the secretion level of TGF-β and TNF-α of iBMDMs transfected with control, si-FOXN3, miR-708-5p mimic or miR-708-5p and FOXN3. Data are presented as mean ± SD (*n* = 3), and statistical significance was assessed by a one-way ANOVA with Tukey’s multiple comparisons test. **P < 0.01; ***P < 0.001.

Given that FOXN3 deficiency promotes macrophage polarization towards the M2 phenotype, we hypothesized that restoring FOXN3 expression in macrophages, particularly in miR-708-5p-expressing cells, would alter the polarization status of these macrophages. To validate this hypothesis, we transfected THP-1 (Mφ) and RAW264.7 cells with flag-FOXN3 stably expressing NC or miR-708-5p. qRT-PCR analysis revealed that, compared with the control, the expression of M2-macrophage markers (Arg1, CD206, IL-10 and TGF-β) were significantly elevated in THP-1 (Mφ) or Raw264.7 cells transfected with the miR-708-5p mimic while the expression of M1-macrophage markers (NOS2 and TNF-α) were decreased, which phenomenon was reversed by re-expression of FOXN3 in corresponding macrophages (Fig. 6A and Supplementary Fig. 6A). Flow cytometry analysis further supported these findings, showing an increased percentage in THP-1 (Mφ) or RAW264.7 cells with elevated expression of CD206 when miR-708-5p was overexpressed, however, the expression level of CD206 was downregulated when FOXN3 was re-expressed (Fig. 6B and Supplementary Fig. 6B). These results indicate that the re-expression of FOXN3 suppresses macrophage polarization towards the M2 phenotype.

To further assess the role of FOXN3 re-expression in macrophages on melanoma growth, A375 cells co-cultured with THP-1 (Mφ) transfected with miR-708-5p exhibited enhanced invasion and migration, which effects were significantly attenuated upon re-expression of FOXN3 in the macrophages (Fig. 6C). Furthermore, we found increased proliferation of A375 cells co-cultured with THP-1 (Mφ) that were transfected with miR-708-5p, which was significantly attenuated when FOXN3 was re-expressed in the macrophages (Fig. 6D). These findings suggest that FOXN3 is indispensable for the promotive effects of miR-708-5p on macrophage M2 polarization and that re-expression of FOXN3 in macrophages can suppress the pro-metastatic effects induced by miR-708-5p, thereby inhibiting melanoma cell proliferation, invasion, and migration.

Previous studies indicate that FOXN3 may be involved in modulating the PI3K/AKT signaling pathway [31]. To further explore the underlying mechanism by which miR-708-5p promotes macrophage polarization by targeting FOXN3, we examined the PI3K/AKT signaling pathway. We found that the treatment with A375-derived exosomes, transfection with miR-708-5p in THP-1 (Mφ) or PBMC-derived macrophages promoted the phosphorylation of mTOR, AKT and PI3K and similar results were obtained from Raw264.7 cells expressing miR-708-5p, while phosphorylation of PI3K, AKT and mTOR were decreased when FOXN3 was re-expressed in corresponding macrophages (Fig. 6E, F and Supplementary Fig. 6C). The enhanced migration and proliferation capabilities of A375 cells induced by macrophages transfected with the miR-708-5p mimic were abolished by LY294002, an inhibitor of the PI3K-AKT signaling pathway (Fig. 6G, H). Furthermore, we stably expressed control, si-FOXN3, miR-708-5p mimic, or co-expressed miR-708-5p mimic and flag-FOXN3 in immortalized bone marrow-derived macrophages (iBMDMs) and examined the secretion of TGF-β and TNF-α by iBMDMs through ELISA. The results suggest that the secretion of TGF-β was increased while that of TNF-α was decreased when iBMDMs were transfected with si-FOXN3 or miR-708-5p mimic, which will be recovered when re-expressing FOXN3 (Fig. 6I). All these findings indicate that miR-708-5p promotes M2 polarization of macrophages by targeting FOXN3 and activating PI3K/AKT pathway.

## Discussion

**Fig. 7:**
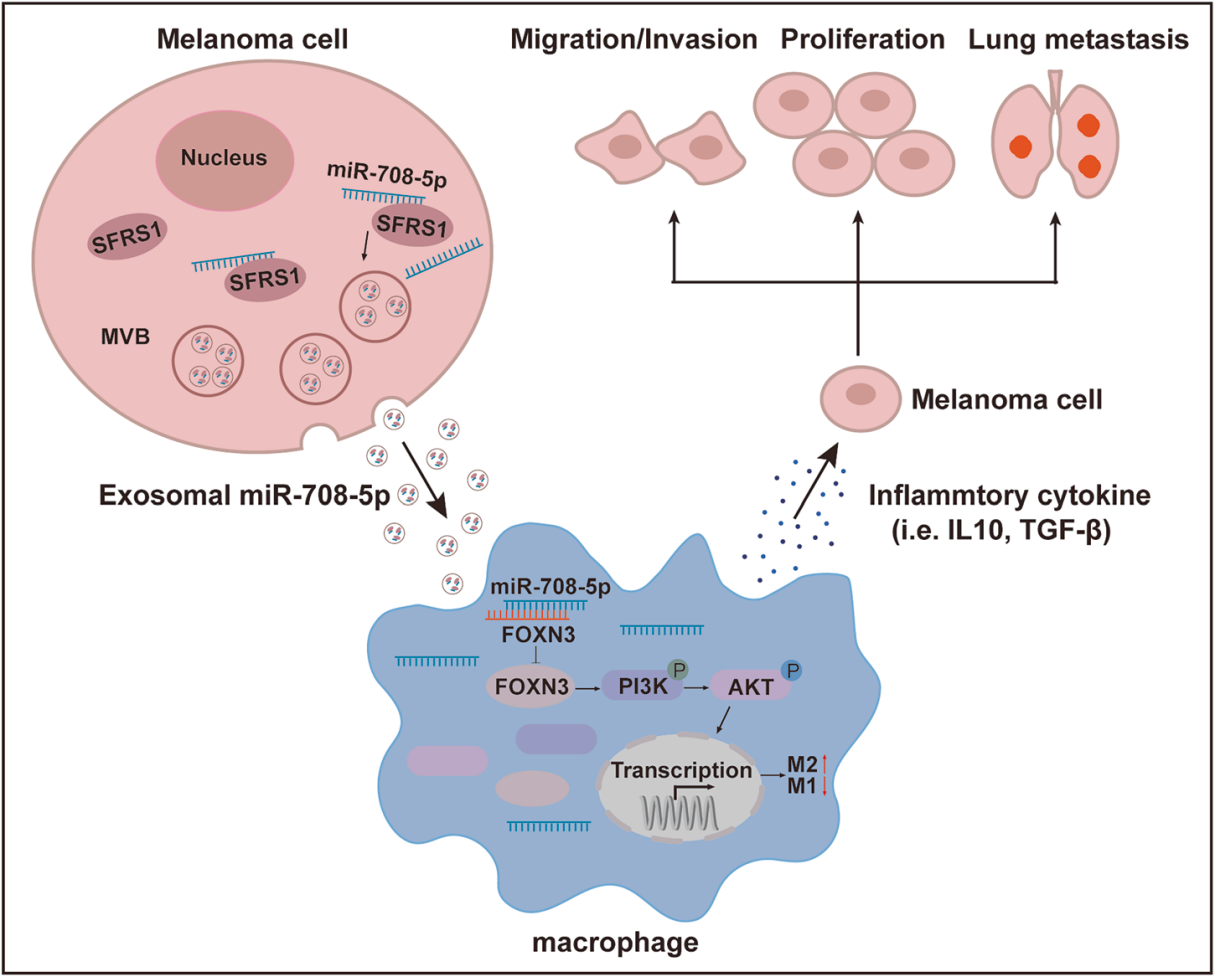
Mechanism raised by this study. A schematic diagram depicting the biological function and mechanism of melanoma-cell-derived miR-708-5p in mediating the M2 polarization of macrophages and promoting melanoma progression and metastasis.

Based on our study, we proposed a molecular mechanism underlying the interaction between melanoma cells and macrophages (Fig. 7). In melanoma cells, miR-708-5p binds to SFRS1 and is subsequently packaged into exosomes. The secreted exosomes containing miR-708-5p internalized by macrophages, in which miR-708-5p directly targets FOXN3 and activates the PI3K/AKT/mTOR pathway, thereby promoting M2 polarization of macrophages. The polarized macrophages secrete immunosuppressive cytokines, such as IL-10 and TGF-β, contributing to the formation of an immunosuppressive tumor microenvironment that enhances the proliferation, invasion, migration and metastasis of melanoma.

Melanoma development is regulated not only by intrinsic properties of tumor cells but also by the surrounding microenvironment, which comprises various immune cell types [32]. Through intercellular communication, these cells contribute to the formation of an immunosuppressive microenvironment, thereby participating in tumor development and metastasis [33, 34]. Accumulating evidences indicate that heterogeneous tumor-associated macrophages significantly impact cancer cell metastasis. The dynamic balance between different subtypes of macrophages may be critical in determining the pro-tumorigenic or anti-tumorigenic effects of the melanoma microenvironment. Emerging evidences underscore the pivotal roles of miRNAs in both the initiation and progression of tumors. Within the highly regulated tumor microenvironment (TME), intercellular communication drives cancer progression and metastasis. Exosome-mediated transfer of molecular cargo has been extensively documented to facilitate tumor-stromal interactions, thereby promoting the formation of an immunosuppressive niche [8, 9]. Our study demonstrated that exosomes derived from melanoma cells rather than other secreted factors induced M2 polarization of macrophages, similar to what have been found in other solid tumors, suggesting that the utilization of exosomes as key vehicles for intercellular communication may be adopted as a conventional mechanism.

miR-708-5p has been implicated in diverse diseases, such as cardiovascular disorders [35, 36], immune responses [37, 38] and cancer [39, 40]. Although miR-708-5p has been extensively studied in diverse contexts, its role in macrophage regulation within the tumor microenvironment remains poorly understood. Our study reveals that miR-708-5p is significantly enriched in melanoma exosomes and promotes M2 polarization after macrophage uptake. This phenotypic shift in macrophages enhances the invasion, migration, EMT, proliferation of melanoma cells and increases lung metastasis. Although we identified miR-708-5p as an important regulatory molecule for macrophage polarization and melanoma development, we could not exclude the possibility that other contents in the A375-derived exosomes may also play roles in these processes.

By screening the candidate RNA-binding proteins (RBPs) [41, 42], we found that the direct binding of miR-708-5p to SFRS1 may be a key step for its entry into exosomes, which provides important clues for us to understand the mechanism of selective miRNA packaging. More interestingly, we found that intracellular accumulation of miR-708-5p will cause elevated apoptosis and reduced proliferation of melanoma cells, indicating that miR-708-5p may serve as a tumor suppressor inside melanoma cells. However, in macrophages miR-708-5p can promote M2 polarization and enhance tumor malignant behavior. This “dual role” phenomenon is noteworthy, which suggests that the function of a single miRNA in different cells can also be different. Tumor cells may promote the formation of an immunosuppressive microenvironment by selectively packaging certain miRNAs—potentially detrimental to themselves—into exosomes, which will be delivered into immune cells and regulate their function, and therefore achieving dual roles of self-protection and microenvironment remodeling. This interesting phenomenon also exists in other tumors. For example, hnRNPA2B1-mediated exosomal transfer of tumor-suppressive miR-184-3p from breast cancer cells to macrophages, which regulate TNBCs progression [43]. In glioma, Tumor-derived exosomes deliver the tumor suppressor miR-3591-3p to induce M2 macrophage polarization and promote glioma progression [44]. These findings indicate that miRNA function is not solely determined by its sequence but is also influenced by cell type and other physiological or pathological conditions.

miRNAs function by binding to the 3’-UTRs of target mRNAs, leading to mRNA degradation or translational repression. This regulatory mechanism can influence various cellular processes, including cancer progression [45]. We found that miR-708-5p targets FOXN3 to activate the PI3K/AKT signaling pathway, which plays a pivotal role in regulating macrophage polarization, influencing their functional phenotypes and subsequent impacts on the tumor microenvironment [46, 47]. FOXN3 has rarely been reported to play a role in macrophage function. In this study, FOXN3 was integrated into the macrophage immune regulatory network for the first time.

In summary, we found that melanoma exosomal miR-708-5p activates macrophages toward the M2 type by targeting FOXN3 and mediating PI3K/AKT pathway activation, which in turn promote melanoma progression. In addition, miR-708-5p functions as a tumor suppressor in melanoma and is selectively sorted into exosomes by SFRS1, ensuring the efficient release of miR-708-5p into the extracellular environment. This study not only enhance the understanding about the biological function of miRNAs, exosomes and the mechanism of melanoma development, but also offer novel insights into melanoma therapy. Engineered exosomes loaded with si-miR-708-5p may be designed, which can specifically target macrophages and reprogram macrophages to M1 type, thus restoring immune activity and inhibiting tumor development.

## Materials and methods

### Cell culture

All the cell lines used in this study were obtained from ATCC, and have been authenticated by STR DNA profiling analysis and routinely tested for Mycoplasma contamination (Beyotime). All cells were maintained at 37 °C, 5% CO_2_ and 100% humidity. HEMa cells were grown in Dermal Cell Basal Media supplemented with Adult Melanocyte Growth Kit components, melanoma cells and 293 T cells were cultured in Dulbecco’s modified Eagle’s medium (DMEM) supplemented with 10% fetal bovine serum (FBS, Sigma). The macrophages, Raw264.7 and BMDM were cultured in Dulbecco’s modified Eagle’s medium (DMEM), and THP-1 cells were cultured in RPMI-1640 medium (Gibco), with all medium supplemented with 10% FBS (Gibco). For all cell culture, 100 U/mL penicillin and 100 μg/mL streptomycin (Cytiva) were supplemented. 50 ng/mL phorbol-12-myristate-13-acetate (PMA; Sigma–Aldrich) was used to treat THP-1 cells for 24 h for M0 macrophages differentiation. For the co-culture assay, HEMa or A375 were plated into a six-well plate with or without 5 μM exosome secretion inhibitor GW4869 (MedChemExpress), THP-1 cells were seeded to 0.4 μm-diameter culture inserts (Corning) and induced into macrophages by PMA. After 24 h, THP1 culture inserts were added above the HEMa or A375 cells for another 48 h.

### Isolation of PBMC-derived macrophage

Peripheral blood mononuclear cells (PBMCs) were isolated from blood samples from healthy donors (Oribiotech) using density gradient centrifugation (Solarbio) followed by APC magnetic beads selection (Biolegend). The purified PBMC were then cultured in RPMI-1640 medium supplemented with macrophage colony-stimulating factor (M-CSF; 20 ng/mL) to drive differentiation into macrophages. All human experiments were approved by the Ethics Committee of Taichang Hospital. Written informed consent was obtained from all participants prior to their involvement in the study.

### Exosome isolation and characterization

For exosome isolation, A375 cells were cultured in DMEM supplemented with 10% FBS which was depleted of exosomes by ultra-centrifugation at 110,000 × *g* at 4 °C for 16 h. When cells reached 80%–90% confluent, the cell culture medium was harvested and centrifuged at 500 g for 10 min at 4 °C, followed by 10,000 × *g* for 30 min at 4 °C to remove residual cells and debris. The cell supernatants were filtered by a 0.22 μm filter and ultracentrifuged at 110,000 × g for 70 min (Beckman Coulter) to collect the pellet and then washed in PBS before centrifugation at 110,000 × *g* for 70 min at 4 °C. Finally, exosomes were resuspended in desired volumes of PBS. Quantification was performed using the BCA Protein Assay Kit (CWBio).

For TEM imaging (Thermo Scientific), 5 μl of exosome suspension was applied to a carbon-coated copper grid for 1 min, followed by removal of excess liquid using a filter paper. Negative staining was performed with 2% uranyl acetate for 1 min incubation before residual stain was removed. After air-drying for 5 min, samples were visualized under an 80 keV transmission electron microscope. Exosome size distribution and concentration were quantified using a ZetaView instrument (Brookhaven). Prior to analysis, samples were diluted to 0.1 mg/ml in PBS and filtered through a 0.22 μm filter to eliminate aggregates. The instrument was preheated for 30 min to stabilize the laser and temperature. The exosome marker proteins were validated by western blotting, as detailed in ‘Western blotting’ of the method section.

### Exosome uptake and treatment

To monitor exosomal trafficking, the PBS-suspended exosomes were stained with CFSE (Invitrogen), a cell membrane labeling agent, at a final concentration of 5 μM for 1 h at room temperature. After staining, the exosomes were washed with PBS to remove the excess dye. THP1(Mφ) macrophages and Raw264.7 cells were seeded into a 35 mm confocal dishes and incubated with CFSE-labeled exosomes for 24 h. Then, the macrophages were fixed with 4% PFA for 20 min, subsequently permeabilized with 0.01% Triton-100 for 15 min. Rhodamine Phalloidin (ABclonal) were used to label cytoskeleton and nuclear staining was performed using DAPI (Sigma-Aldrich). Images were acquired using the Zeiss 980. For in vitro cell treatment, 40 μg of exosomes was added per well of a 12-well plate with 1 ml medium. Cells were harvested after 24 h for RNA extraction or after 48 h for western blot.

### Plasmid construction and lentivirus preparation

The human FOXN3 cDNA was cloned into a pHAGE lentiviral vector with flag tag, which is commonly used for stable gene expression in mammalian cells. In order to generate lentiviral particles, the lentiviral vector (containing flag-FOXN3), psPAX2 (a packaging plasmid), and pMD2.G (an envelope plasmid) were co-transfected into HEK293T cells for 48 h. The lentiviral particles infection was conducted with 8 μg/mL polybrene (Sigma-Aldrich) to improve transduction efficiency. All reagents used in this study are listed in Supplementary Table S1.

### Cell transfection

The miR-708-5p mimics/inhibitors and the FOXN3 siRNAs were designed and synthesised from GenePharma (Shanghai). LipofectamineTM 3000 Transfection Reagent (Thermo) was used to transfect the miRNA mimics, inhibitors and siRNAs into cells according to the manufacturer’s protocols. Cells were harvested for RNA extraction after 24 h. For protein sample collection, the cell culture medium was replaced with fresh medium for an additional 24 h incubation. All sequences are listed in Supplementary Table S2.

### RNA extraction and qRT-PCR

Total RNA from cells was extracted using TRIzol reagent (Invitrogen, 15596026). RNA (1 μg per sample) was reverse transcribed into complementary DNA (cDNA) using HiScript III RT SuperMix (Vazyme). Quantstudio5 real-time PCR system (Thermo) was used to perform qRT-qPCR using ChamQ Universal SYBR qPCR Master Mix (Vazyme). All standards and samples were assayed in triplicate. All primers were designed using PrimerBank and sequences are listed in Supplementary Table S3. All raw data were provided in Supplementary Materials Raw Data of Real-time PCR

### RNA sequencing

HEMa exosomes (control) or A375 exosomes were incubated with THP1 (Mφ) macrophages for 24 h. Subsequently, the macrophages were washed with pre-cold PBS and subjected to RNA sequencing by the company Novogene.

### Western blotting

Total cellular protein and exosomal protein were lysed in using NP40 lysis buffer. Loading buffer was added to the protein lysates and boiled for 15 min at 95 °C. Protein extracts were separated by SDS–PAGE gels (GenScript) and transferred onto 0.22 μm polyvinylidene fluoride (PVDF) membranes (Millipore). After blocked with 5% skimmed milk for 2 h at room temperature (Sigma–Aldrich), membranes were incubated with primary antibodies (Supplementary Table S4) overnight at 4 °C and then washed with TBST for 3 times. After washing, membranes were incubated with HRP-conjugated secondary antibodies (Jackson) for 1 h at room temperature and washed with TBST for 3 times. Protein bands were visualized by enhanced chemiluminescence (ECL; ABclonal) using Amersham Imager 680 system (Bioke).

### Flow cytometry

THP-1 cells or Raw264.7 cells were harvested, washed with PBS, and fixed with 2% paraformaldehyde (PFA) at 4 °C. The cells were then blocked with CD16/32 or TruStain (BioLegend) to minimize non-specific binding. Following blocking, cells were washed and resuspended in flow cytometry buffer and stained with PE-CD206 or APC-CD86 on ice for 20 min. IgG isotype controls were used as negative controls. Finally, samples were washed, resuspended in flow cytometry buffer, and then subjected to flow cytometric analysis using a BD FACS instrument. Data were analyzed using FlowJo software (TreeStar).

### Dual-luciferase reporter assay

Wild-type or mutant FOXN3 3’-UTR containing the binding sites of miR-708-5p were cloned into the psiCHECK-2 luciferase vector. Cells were seeded into 24-well plates and co-transfected with the cloned plasmids and miR-708-5p mimics when cells reached ~50% confluency. Then. 48 h after transfection, cells were lysed with lysis buffer, harvested and centrifuged at 12,000 × g for 3 min. Cell supernatant was harvested and the luciferase activity was measured according to the manufacturer’s instructions (Promega).

### Cell migration and invasion

According to previous study [48], 24-well plates with 8 μm pore size Transwell inserts (Corning Life Science) were used for both migration and invasion assays. For the invasion assay, Matrigel was applied to the upper surface of the Transwell insert. In brief, Tumor cells (3 × 10^4^) suspended in 200 μL serum-free medium were seeded into the upper chamber, and conditioned macrophages were added to the bottom chamber containing 800 μl medium supplemented with 10% FBS. After 48 h of co-culture, tumor cells were fixed with 4% paraformaldehyde (PFA) for 20 min and stained with 0.1% crystal violet (Biosharp) for 30 min, and non-migrating or non-invading cells in the upper chamber were carefully removed using a cotton swab. Three random visual fields were selected, and the number of migrated or invaded cells was counted under a light microscope.

### Cell proliferation

Condition medium from macrophages were added into A375 cells for 24 h, then cells were added with EdU reagent and incubated at 37 °C for 2 h. Later, cells were fixed with 4% paraformaldehyde (PFA) for 20 min and stained according to the manufacturer’s instructions (Beyotime).

### RNA-binding protein immunoprecipitation (RIP) assay

Cells or exosomes were lysed using lysis buffer (supplemented with RNase inhibitor and protease inhibitor cocktail). Protein A beads were first incubated with anti-SFRS1 or IgG (Control) overnight at 4 °C. Next, the lysate was incubated with beads-antibody complex for 4 h at 4 °C. After washing the beads five times, RNA purification and extraction were performed. The isolated RNA was reverse transcribed and performed qRT-PCR.

### miRNA pull down

Cells transfected with biotinylated miR-708-5p or its mutant were lysed using lysis buffer supplemented with RNase inhibitor and protease inhibitor cocktail. Streptavidin magnetic beads were then added to the lysate and incubated overnight at 4 °C. Following five washes, elute the binding protein with elution buffer and subsequently analyzed by western blotting for protein identification.

### Animal experiment

To examine the effects of exosome or miR-708-5p-treated macrophage on tumor growth in vivo, fifteen BALB/c nude mice (male, 6–8 weeks old) were purchased from Shanghai Nanfang Model Biotechnology (Shanghai, China) and were randomly divided into 3 groups (*n* = 5 per group). Approximately 5 × 10^6^ A375 cells and 1 × 10^6^ conditioned macrophages (stimulated by PBS, A375-Exos, or transfected by miR-NC and miR-708-5p mimics) were subcutaneously injected into the right flank of mice to establish a xenograft model. The tumor volume was measured every 2 or 3 days. All nude mice were sacrificed when the tumor reached a defined volume, and the transplanted tumors were isolated for further experiments. Furthermore, to explore the role of miR-708-5p on melanoma growth, A375 were first transfected with NC or miR-708-5p mimics, then the cells (5 × 10^6^ cells per mouse) were injected into the right flank of mice. The tumor volume was measured as above.

To examine the effects of the conditioned macrophages on tumor metastasis in vivo, Clodronate liposomes (Yeasen) were injected into each mouse for macrophage depletion. THP1 (1 × 10^6^ cells) stimulated with PBS, exosomes derived from A375 or miR-708-5p were injected into the mice via tail-vein interval for 3 days. luciferase-labeled A375 cells (5 × 10^6^ cells per mouse) were intravenously injected into the mice. Prior to imaging, each mouse was injected with D-Luciferin (150 mg/kg, Yeasen). Tumor metastasis was measured by in vivo bioluminescent imaging (Biospace Lab). Then all mice were euthanized. The lung tissues of all mice were subjected to H&E staining to examine the metastases sites. The implementation of the experiment was completed under double-blind conditions. All animal procedures were carried out in accordance with the Guide for the Care and Use of Laboratory Animals with the approval of Laboratory Animal Ethics Committee.

### Statistical analysis

The data were visualized by GraphPad Prism10 software (GraphPad Software, USA). Results are expressed as the mean ± SD. Statistical significance was evaluated using a one-way ANOVA with Tukey’s multiple comparisons test and unpaired student’s *t* test, *P < 0.05, **P < 0.01, and ***P < 0.001 indicate a significant difference, *P* > 0.05 was considered not significant and was denoted by “N.S.”

## Supplementary information

Supplementary information

Original Data

## Data Availability

The miRNA-seq data used in this study are available from the corresponding author upon reasonable request.
