# Supplementary material for: Melanoma exosomal miR-708-5p promotes macrophage M2 polarization and cancer metastasis
Source: Cell Death Dis. 2026 Mar 24;17(1):346. doi: 10.1038/s41419-026-08597-1 (PMC13039904; doi:10.1038/s41419-026-08597-1)
Supplement: Supplementary file 1 — Supplementary information [file 41419_2026_8597_MOESM1_ESM.docx]

Supplementary Information for

**Melanoma exosomal miR-708-5p promotes macrophage M2 polarization and cancer metastasis**

**Meng Xu*, Bincan He*, Xiaofeng Zhou**, **Li Shu^#^ & Dan Ma^#^**

∗To whom correspondence should be addressed: D. Ma (madan@westlake.edu.cn), L. Shu (shuli@westlake.edu.cn)

This file includes:

Supplementary Figs. 1-6

Supplementary Tables 1-4


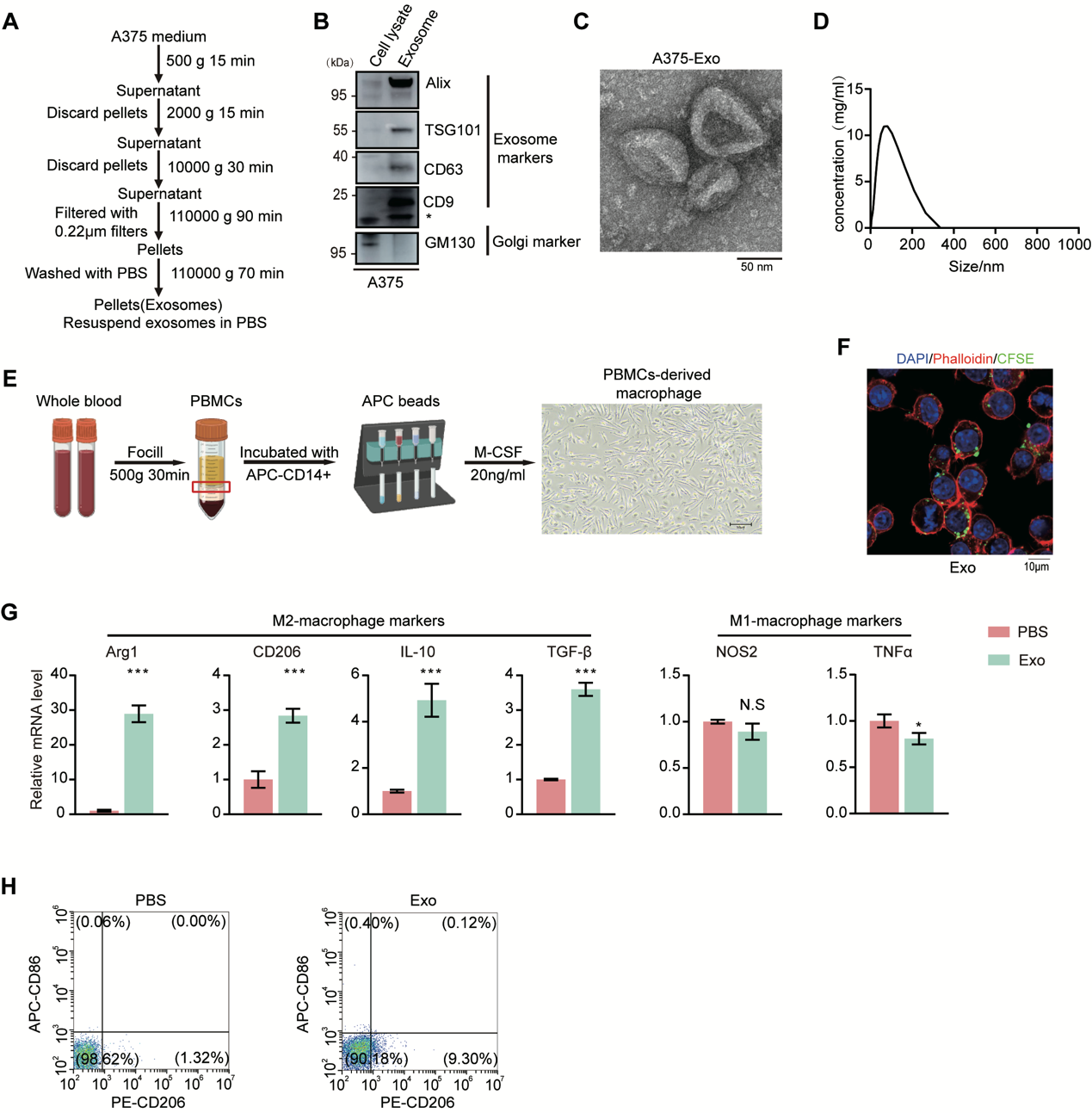


**Supplementary Figure 1. Melanoma-derived exosomes induce M2 polarization of macrophages. (A)** Schematic diagram of the exosome isolation procedure. **(B)** Western blot detection of exosome markers and a Golgi marker in A375 cell lysates and isolated exosomes. **(C)** Negative staining result of isolated exosomes. Scale bar, 50 nm. **(D)** Dynamic Light Scattering analysis of isolated exosomes. **(E)** Schematic diagram of PBMC-derived macrophages isolation and purification procedure. **(F)** Representative immunofluorescence staining of phalloidin labeled cytoskeleton (red) and CFSE labeled B16F10-exosomes (green) for Raw264.7 cells with exosome treatment. Scale bar, 10 μm. **(G)** Analysis using qRT-PCR of M2 markers (Arg1, CD206, IL10, TGF-β) and M1 markers (NOS2, TNF-α) in Raw264.7 cells treated with purified B16F10 exosomes for 24 h. Raw264.7 cells treated with PBS was set as the negative control. Data are presented as mean ±SD (n=3), and statistical significance was assessed by an unpaired student’s t-test. **P* < 0.05; ****P* < 0.001. N.S, not significant. **(H)** Representative dot plots from flow cytometry analysis of the proportion of CD206^+^ macrophages in Raw264.7 cells with or without B16F10-exosome treatment.

**
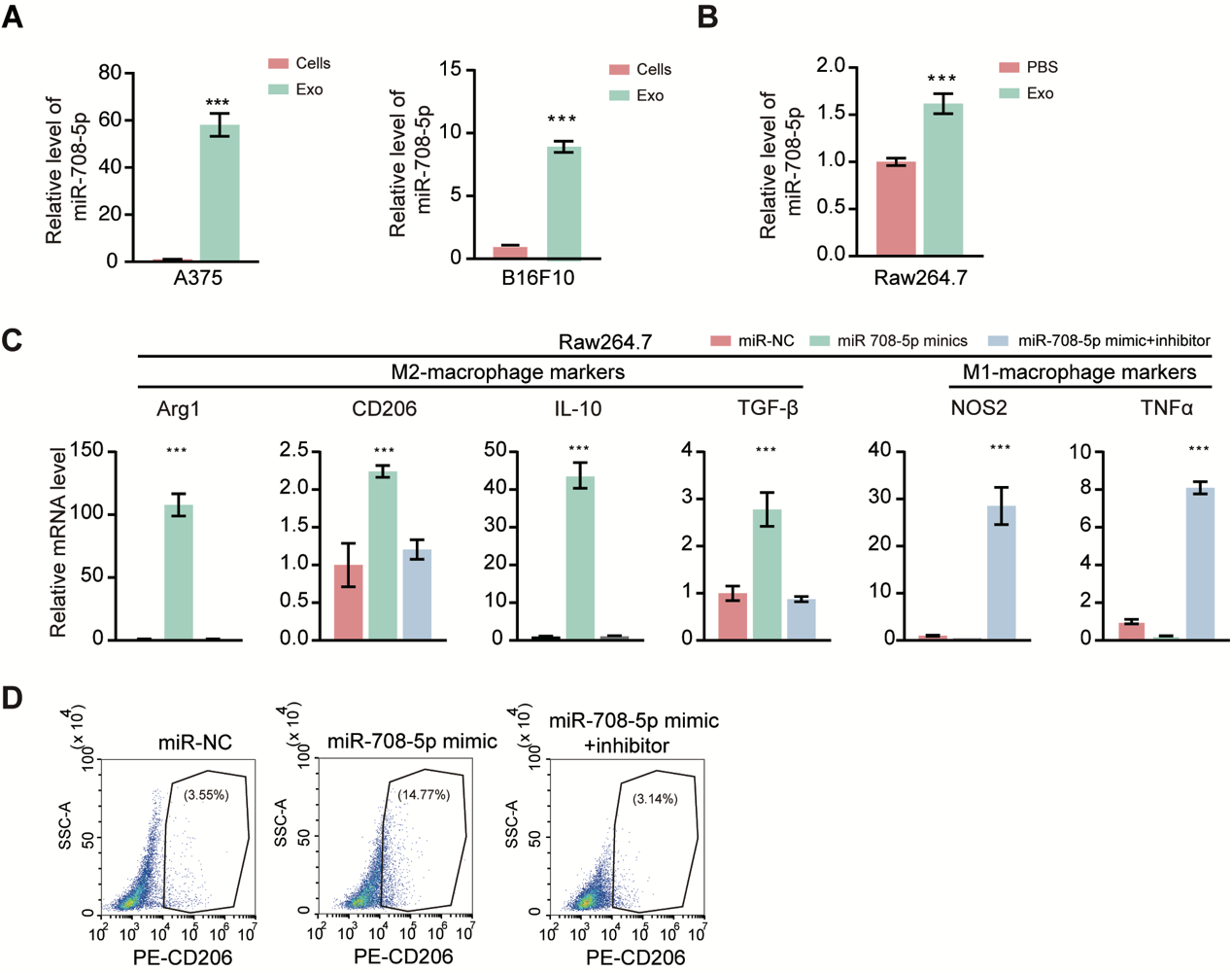
**

**Supplementary Figure 2. Exosomal miR-708-5p promotes M2 polarization in macrophages. (A)** Relative level of miR-708-5p in intracellular or exosomes of A375 or B16F10. Data are presented as mean ±SD (n=3), and statistical significance was assessed by an unpaired student’s t-test. ****P* < 0.001.(**B**) Relative level of miR-708-5p in Raw264.7 macrophages treated with PBS or B16F10 derived exosomes. **(C)** Relative mRNA level of M2 markers (Arg1, CD206, IL10, TGF-β) and M1 markers (NOS2, TNF-α) in RAW264.7 transfected with miR-708-5p mimic, miR-708-5p inhibitor or miR-NC. **(D)** Flow cytometry results of the proportion of CD206^+^ macrophages in Raw264.7 transfected with miR-708-5p mimic, miR-708-5p inhibitor or miR-NC.


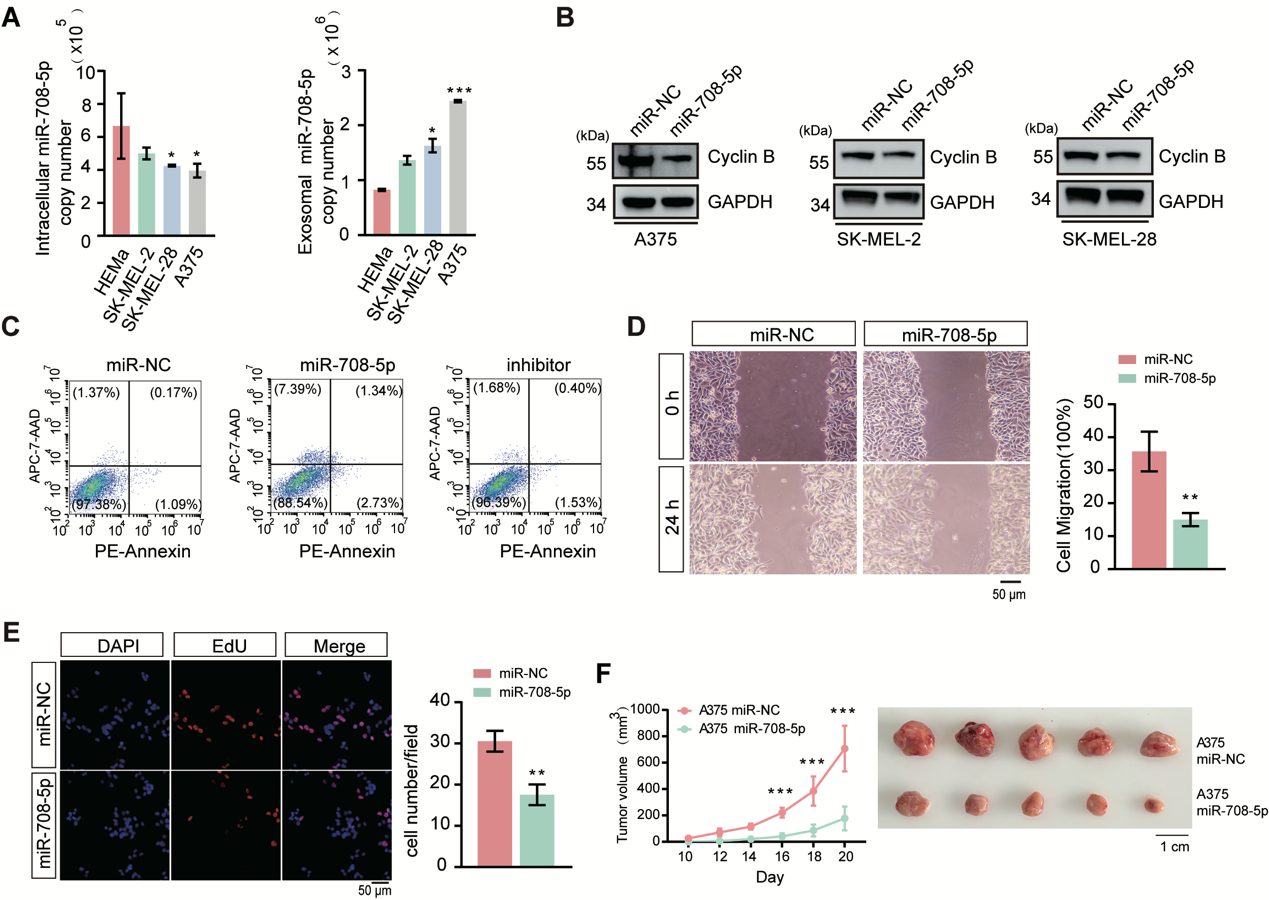


**Supplementary Figure 3. miR-708-5p function as a tumor suppressor in melanoma(A)** Intracellular or exosomal copy number of miR-708-5p in HEMa cells, SK-MEL-2 cells, SK-MEL-28 cells and A375 cells. Data are presented as mean ±SD (n=3), and statistical significance was assessed by a one-way ANOVA with Tukey’s multiple comparisons test. **P* < 0.05; ***P* < 0.01; ****P* < 0.001. **(B)** Western blot analysis of Cyclin B in A375 cells, SK-MEL-2 cells and SK-MEL-28 cells transfected with miR-708-5p mimic. **(C)** Flow cytometry analysis of cell apoptosis markers in A375 cells transfected with miR-708-5p mimic, miR-708-5p inhibitor or miR-NC. **(D)** Migration capability of A375 cells transfected with miR-NC or miR-708-5p mimic was determined by the wound-healing assay. **(E)** Cell proliferation profiles of A375 cells transfected with miR-NC or miR-708-5p mimic and the quantification of cells that were active in proliferation. Scale bar, 50 μm. **(F)** Tumor volume changes in each group were recorded every two days (left, n=5). Statistical significance was determined using two-way ANOVA test. ****P* < 0.001. On day 20, five mice from each group were sacrificed for tumor isolation and comparison (right).


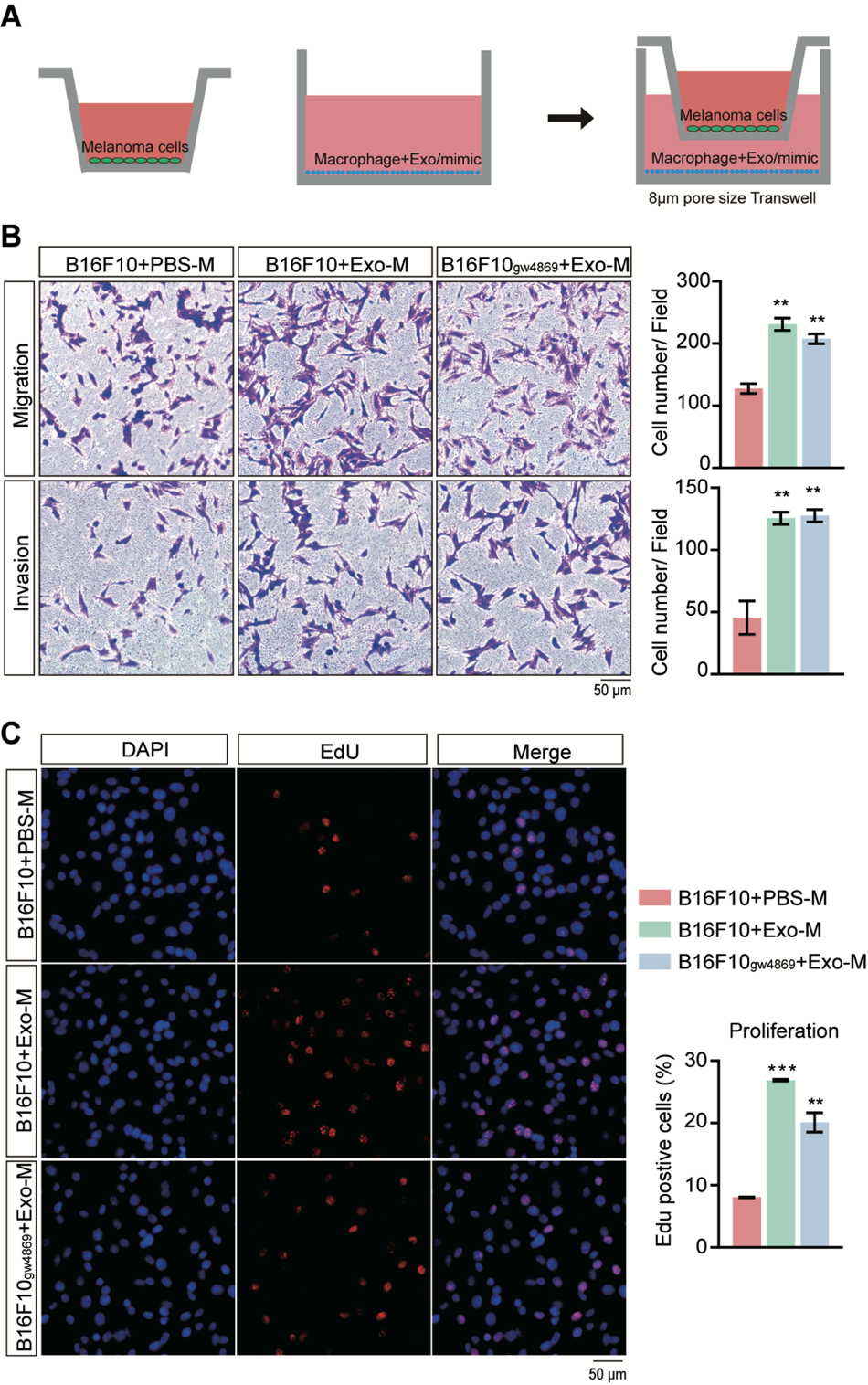


**Supplementary Figure 4. Macrophage induced by melanoma-derived exosomes promote melanoma progression in vitro. (A)** An illustration of the transwell system for co-culturing of melanoma cells with macrophages using a transwell chamber with 8 μm pore size. **(B)** Invasion and migration capabilities of B16F10 cells (with or without GW4869 treatment) co-cultured with conditioned macrophages (PBS-M, Raw264.7 incubated with PBS; Exo-M, Raw264.7 incubated with B16F10-derived exosomes) were determined by the transwell co-culture system. Scale bar, 50 μm. Cell numbers are presented as mean ±SD, and statistical significance was assessed by an unpaired student’s t-test. ****P* < 0.001. (**C)** Proliferation profiles of B16F10 cells (with or without GW4869 treatment) that were incubated with the supernatants of macrophages pre-treated with PBS or exosomes were evaluated by EdU assay. Scale bar, 50 μm. Percentage of EdU-positive cells are presented as mean ±SD, and statistical significance was assessed by an unpaired student’s t-test. ***P*< 0.01.


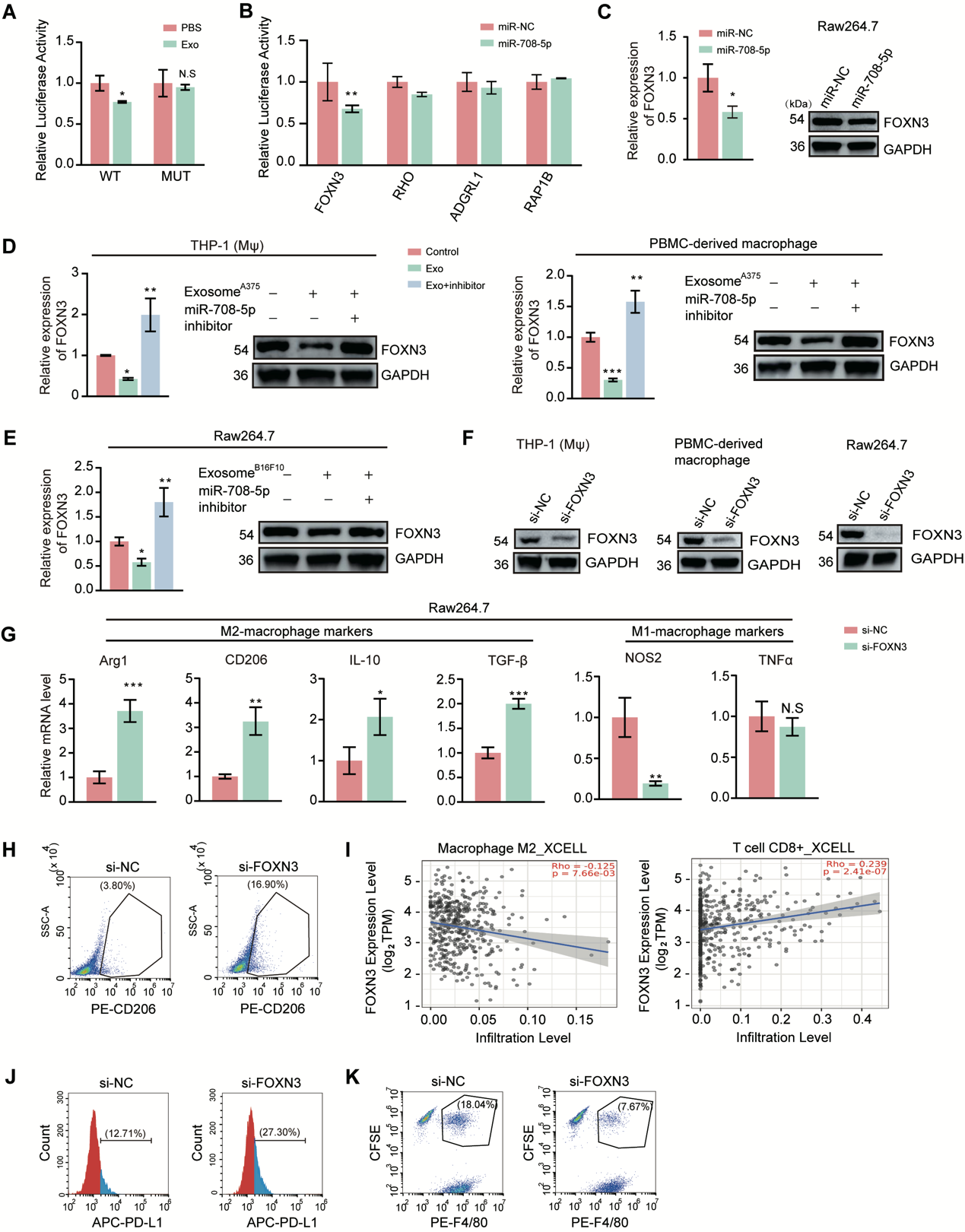


**Supplementary Figure 5. miR-708-5p targets FOXN3 in macrophage to regulate its function. (A)** Relative luciferase activity with the presence of the FOXN3 luciferase reporters (WT and MUT) were performed in cells treated with PBS or exosomes. Data are presented as mean ±SD, and statistical significance was assessed by two-way ANOVA test. **P* < 0.05. **(B)** Relative luciferase activities of luciferase reporters in cells transfected with miR-NC or miR-708-5p mimic. Data are presented as mean ±SD, and statistical significance was assessed by two-way ANOVA test. **P* < 0.05. **(C)** Relative mRNA level and western blot examination of FOXN3 in Raw264.7 cells transfected with miR-NC or miR-708-5p mimic. Data are presented as mean ±SD (n=3), and statistical significance was assessed by an unpaired student’s t-test. **P* < 0.05. **(D-E)** Relative mRNA level and western blot examination of FOXN3 in THP-1 (Mφ), PBMC-derived macrophages and Raw264.7 treated with PBS or exosomes and miR-708-5p inhibitor. Data are presented as mean ±SD (n=3), and statistical significance was assessed by a one-way ANOVA with Tukey’s multiple comparisons test. ***P* < 0.01; ****P* < 0.001. **(F)** Western blot examination of the FOXN3 knockdown efficiency in THP-1 (Mφ), PBMC-derived macrophages and Raw264.7 cells. **(G)** Relative mRNA level of M2 and M1 markers in Raw264.7 transfected with si-NC or si-FOXN3. Data are presented as mean ±SD (n=3), and statistical significance was assessed by an unpaired student’s t-test. **P* < 0.05; ***P* < 0.01; ****P* < 0.001. **(H)** Flow cytometry analysis of the proportion of CD206^+^ macrophages in Raw264.7 transfected with si-NC or si-FOXN3. **(I)** Analysis of the relationship between FOXN3 expression level and M2 phenotype of macrophages, and T cell infiltration in melanoma using the TIMER2.0 server. **(J)** Flow cytometry analysis of the proportion of PD-L1^+^ macrophages in THP-1 (Mφ) transfected with si-NC or si-FOXN3. **(K)** Flow cytometry analysis of the proportion of apoptotic A375 cells engulfed by THP-1 (Mφ) transfected with si-NC or si-FOXN3.


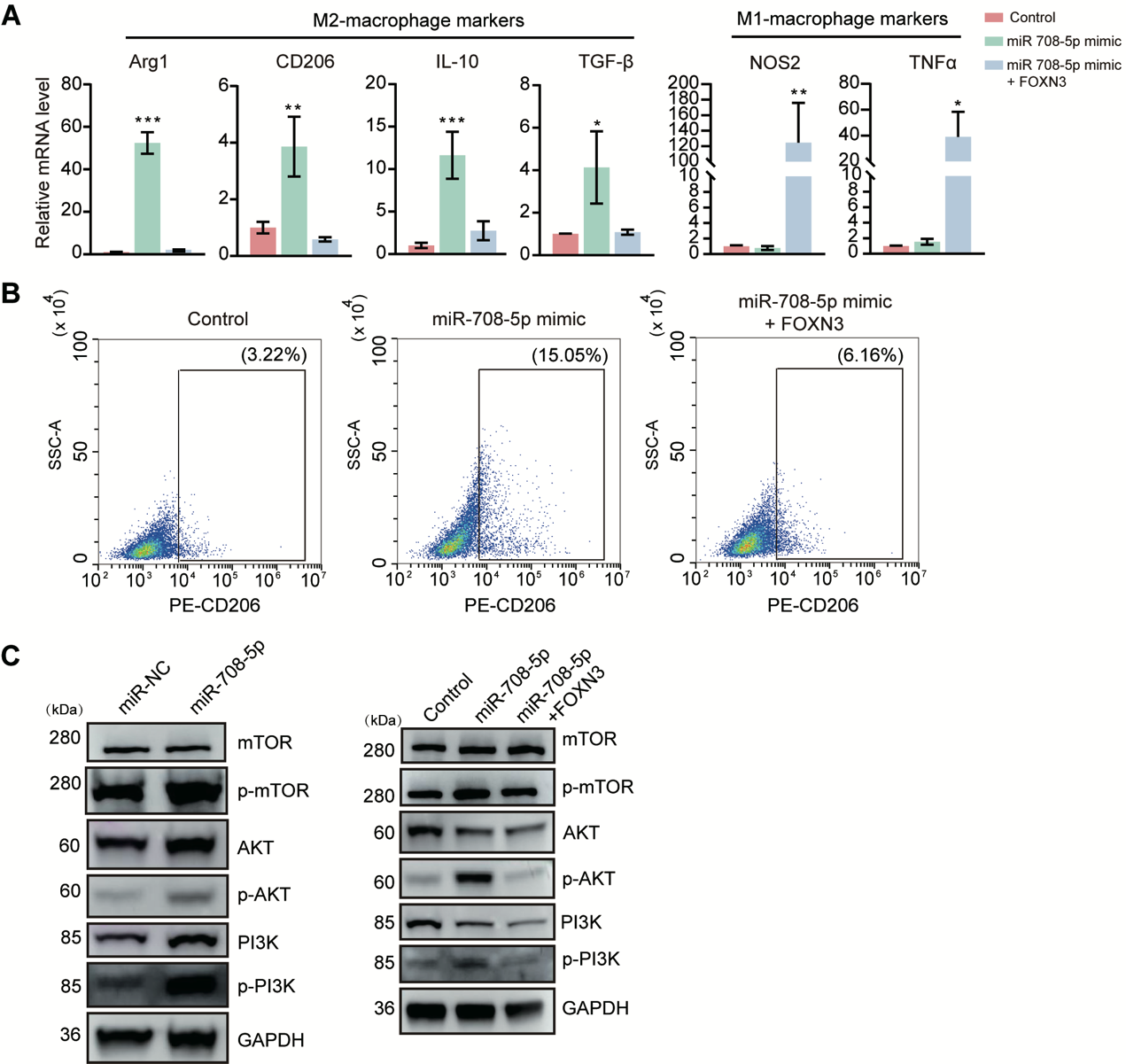


**Supplementary Figure 6. FOXN3 promotes M2 polarization of macrophage through upregulating the PI3K-AKT signaling pathway. (A)** Relative mRNA level of M2 markers (Arg1, CD206, IL10, TGF-β) and M1 markers (NOS2, TNF-α) in Raw264.7 transfected with NC, miR-708-5p or miR-708-5p following with Flag-FOXN3. Data are presented as mean ±SD (n=3), and statistical significance was assessed by a one-way ANOVA with Tukey’s multiple comparisons test. **P* < 0.05; ***P* < 0.01; ****P* < 0.001. **(B)** Flow cytometry analysis of the proportion of CD206^+^ macrophages in Raw264.7 cells treated in the same way as described in **A**. (**C)**, Western blot detection of the expression level of the related proteins in PI3K/AKT/mTOR pathway in Raw264.7 cells transfected with NC, miR-708-5p or miR-708-5p following with Flag-FOXN3.

**Supplementary Table 1. Reagents used in this study.**

| **Reagent** | **Identifier** | **Company** | **Work concentration** | |
| --- | --- | --- | --- | --- |
| Polybrene  CFSE | 107689  65-0850-84 | Sigma–Aldrich  Invitrogen | | 8 μg/ml  5 μM |
| GW4869 | HY-19363 | MedChemExpress | | 5 μM |
| PMA | 16561-29-8 | Sigma–Aldrich | | 50 ng/ml |
| DAPI | D5942 | Sigma–Aldrich | | 1 μg/ml |
| TRIzol | 15596026 | Invitrogen | |  |
| LY294002 | #5536 | Cell Signaling Technology | | 10 μM |
| D-Luciferin | 40901ES01 | Yeasen | | 150 mg/kg |
| Clodronate Liposomes  Rhodamine  Phalloidin  LipofectamineTM 3000 Transfection Reagent  Animal-Free Recombinant Human M-CSF | 40337ES08  RM02835  L3000015  AF-300-25-10U | Yeasen  ABclonal  Thermo Fisher  Dakewe | | 1:200  20 ng/ml |

**Supplementary Table 2. Sequences for siRNAs, miRNA mimics and inhibitors.**

| **Name** | **From 5' to 3'** |
| --- | --- |
| si-NC sense  si-NC antisense  si-FOXN3 sense  si-FOXN3 antisense  mimics NC sense  mimics NC antisense  miR-708-5p mimic sense  miR-708-5p mimic antisense  inhibitor NC  miR-708-5p inhibitor  si-SFRS1-1 sence  si-SFRS1-1 antisence  si-SFRS1-2 sence  si-SFRS1-2 antisence | UUCUCCGAACGUGUCACGUTT  ACGUGACACGUUCGGAGAATT  GGUCCUGUUUGAAUAACAUTT  AUGUUAUUCAAACAGGACCTT  UUUUCCGAACGUGUCACGUTT  ACGUGACACGUUCGGAGAATT  AAGGAGCUUACAAUCUAGCUGGG  CAGCUAGAUUGUAAGCUCCUUUU  CAGUACUUUUGUGUAGUACAA  CCCAGCUAGAUUGUAAGCUCCUU  ACAUAAGAUGAUUGGUGACTT  GUCACCAAUCAUCUUAUGUTT  GCAUCUACGUGGGUAACUUTT  AAGUUACCCACGUAGAUGCTT |

**Supplementary Table 3. Primers for qRT-PCR.**

| **Primer** | **From 5' to 3'** |
| --- | --- |
| Human *GAPDH*-F  Human *GAPDH*-R  Human *CD206*-F  Human *CD206*-R  Human *Arg1*-F  Human *Arg1*-R  Human *IL10*-F  Human *IL10*-R  Human *TGFβ*-F  Human *TGFβ*-R  Human *NOS2*-F  Human *NOS2*-R  Human *TNFα*-F  Human *TNFα*-R  Mouse *Gapdh*-F  Mouse *Gapdh*-R  Mouse *Cd206*-F  Mouse *Cd206*-R  Mouse *Arg1*-F  Mouse *Arg1*-R  Mouse *Il10*-F  Mouse *Il10*-R  Mouse *Tgf-β*-F  Mouse *Tgf-β*-R  Mouse *Nos2*-F  Mouse *Nos2*-R  Mouse *Tnf-α*-F  Mouse *Tnf-α*-R  Human *FOXN3*-F  Human *FOXN3*-R  Mouse *Foxn3*-F  Mouse *Foxn3*-R  Human *SFRS1*-F  Human *SFRS1*-R  Human *PD-L1*-F  Human *PD-L1*-R  Mouse *Pd-l1*-F  Mouse *Pd-l1*-R  miR-708-5p | ACAGTCCATGCCATCACTGCC  GCCTGCTTCACCACCTTCTTG  GGGTTGCTATCACTCTCTATGC  TTTCTTGTCTGTTGCCGTAGTT  CTCCAAGCCAAAGTCCTTAGAG AGGAGCTGTCATTAGGGACATC  GACTTTAAGGGTTACCTGGGTTG  TCACATGCGCCTTGATGTCTG  TACCTGAACCCGTGTTGCTC  GTTGCTGAGGTATCGCCAG  CAGAGGATGGCAGTCTGTTTC  CTCAAGAGCACTGGATCTCAG  TGACAAGCCTGTAGCCC  CCCTTGAAGAGGACCTGG  TGAAGCAGGCATCTGAGGG  CGAAGGTGGAAGAGTGGGAG  CTCTGTTCAGCTATTGGACGC  CGGAATTTCTGGGATTCAGCTTC  CTCCAAGCCAAAGTCCTTAGAG  AGGAGCTGTCATTAGGGACATC  CTTACTGACTGGCATGAGGATCA  GCAGCTCTAGGAGCATGTGG  AAGTTGGCATGGTAGCCCTT  GCCCTGGATACCAACTATTGC  CAGAGGACCCAGAGACAAGC  TGCTGAAACATTTCCTGTGC  CCTCCCTCTCATCAGTTCTA  ACTTGGTGGTTTGCTACGAC  TCGTTGTGGTGCATAGACCC  GTGGACCTGATGTGCTTTGATA  TGCCCGACATCCGATTAGAAG  CTAAGGACCGACTCCCCAAAG  CCGCAGGGAACAACGATTG  GCCGTATTTGTAGAACACGTCCT  TGGCATTTGCTGAACGCATTT  TGCAGCCAGGTCTAATTGTTTT  GCTCCAAAGGACTTGTACGTG  TGATCTGAAGGGCAGCATTTC  AAGGAGCUUACAAUCUAGCUGGG |

**Supplementary Table 4. Antibodies used in this study.**

| **Antibody** | **Identifier** | **Company** | **MW(kDa)** |
| --- | --- | --- | --- |
| GAPDH | #A19056 | ABclonal | 36 |
| PI3K | #4257 | Cell Signaling Technology | 85 |
| p-PI3K | #4228 | Cell Signaling Technology | 85 |
| AKT | #4691 | Cell Signaling Technology | 60 |
| p-AKT | #4060 | Cell Signaling Technology | 60 |
| mTOR | #2983 | Cell Signaling Technology | 289 |
| p-mTOR | #5536 | Cell Signaling Technology | 289 |
| FOXN3 | #25399-1-AP | Proteintech | 54 |
| PD-L1 | #66248-1-lg | Proteintech | 45-50 |
| SFRS1 | #12929-2-AP | Proteintech | 27 |
| CyclinB1 | #12231 | Cell Signaling Technology | 55 |
| N-cadherin | #22018-1-AP | Proteintech | 130 |
| Vimentin | A19607 | ABclonal | 57 |
| MMP7 | A20701 | ABclonal | 20-28 |
| CD63 | #sc-5275 | Santa Cruz | 60 |
| CD9 | #D8O1A | Cell Signaling Technology | 22-35 |
| TSG101 | #MA1-23296 | Thermo Fisher | 50 |
| Alix | #E6P9B | Cell Signaling Technology | 95 |
| Secondary antibody | #111-035-003  #115-035-003 | Jackon |  |
