# Supplementary figures and images for: Melanoma exosomal miR-708-5p promotes macrophage M2 polarization and cancer metastasis
Source: Cell Death Dis. 2026 Mar 24;17(1):346. doi: 10.1038/s41419-026-08597-1 (PMC13039904; doi:10.1038/s41419-026-08597-1)

**Raw Data of Real-time PCR
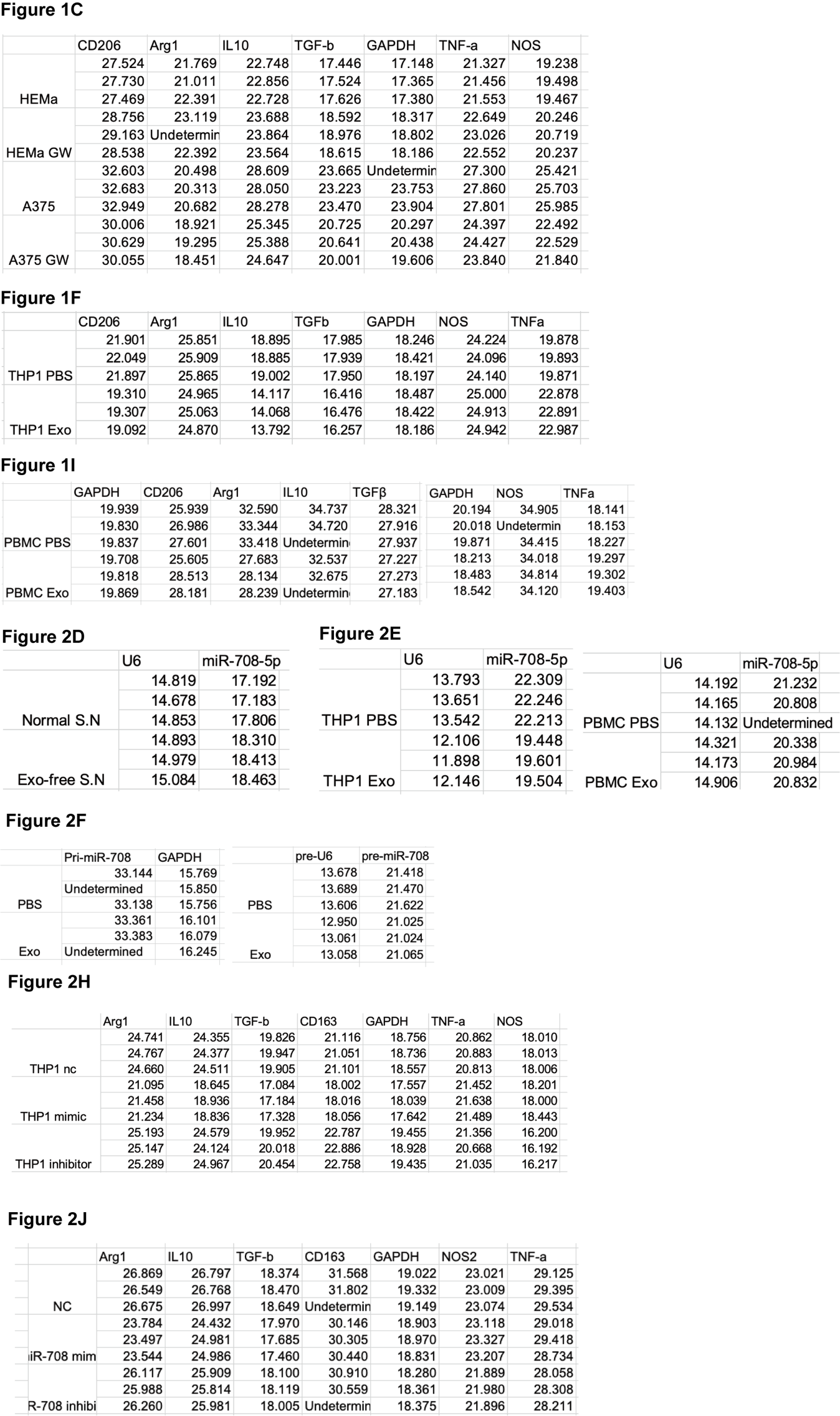
**

**
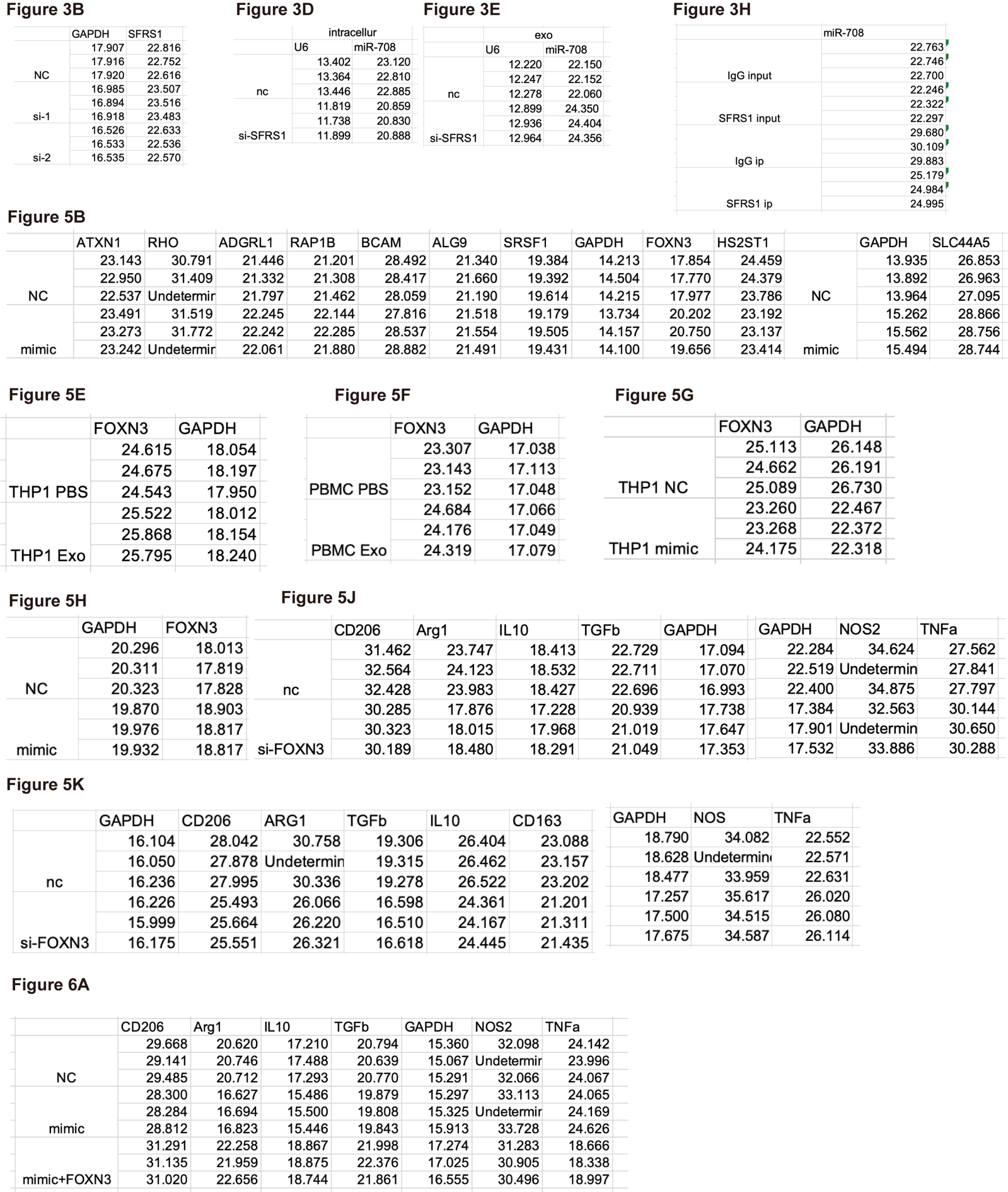
**

**
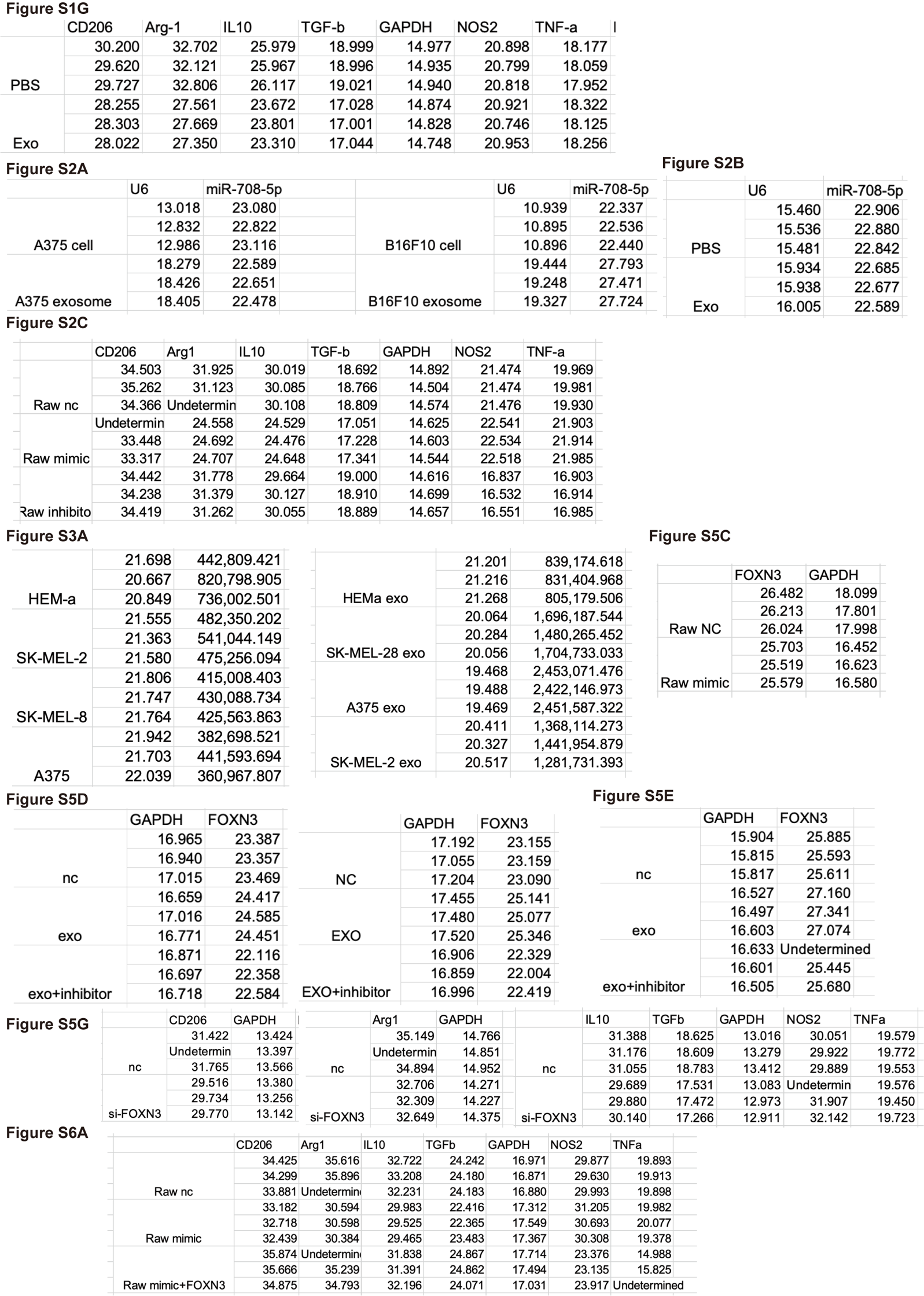
**

**Original Western Blots**

**
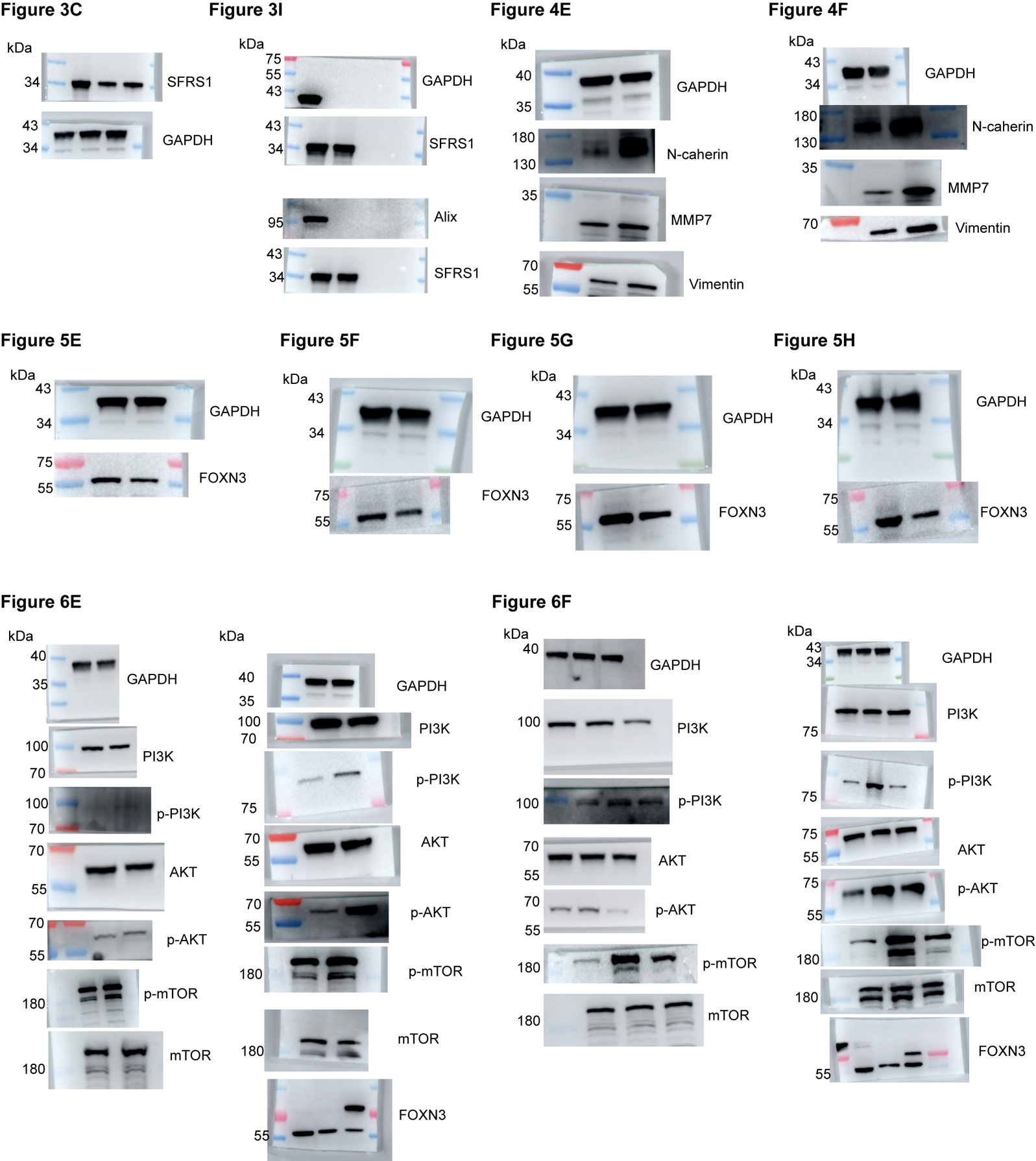
**


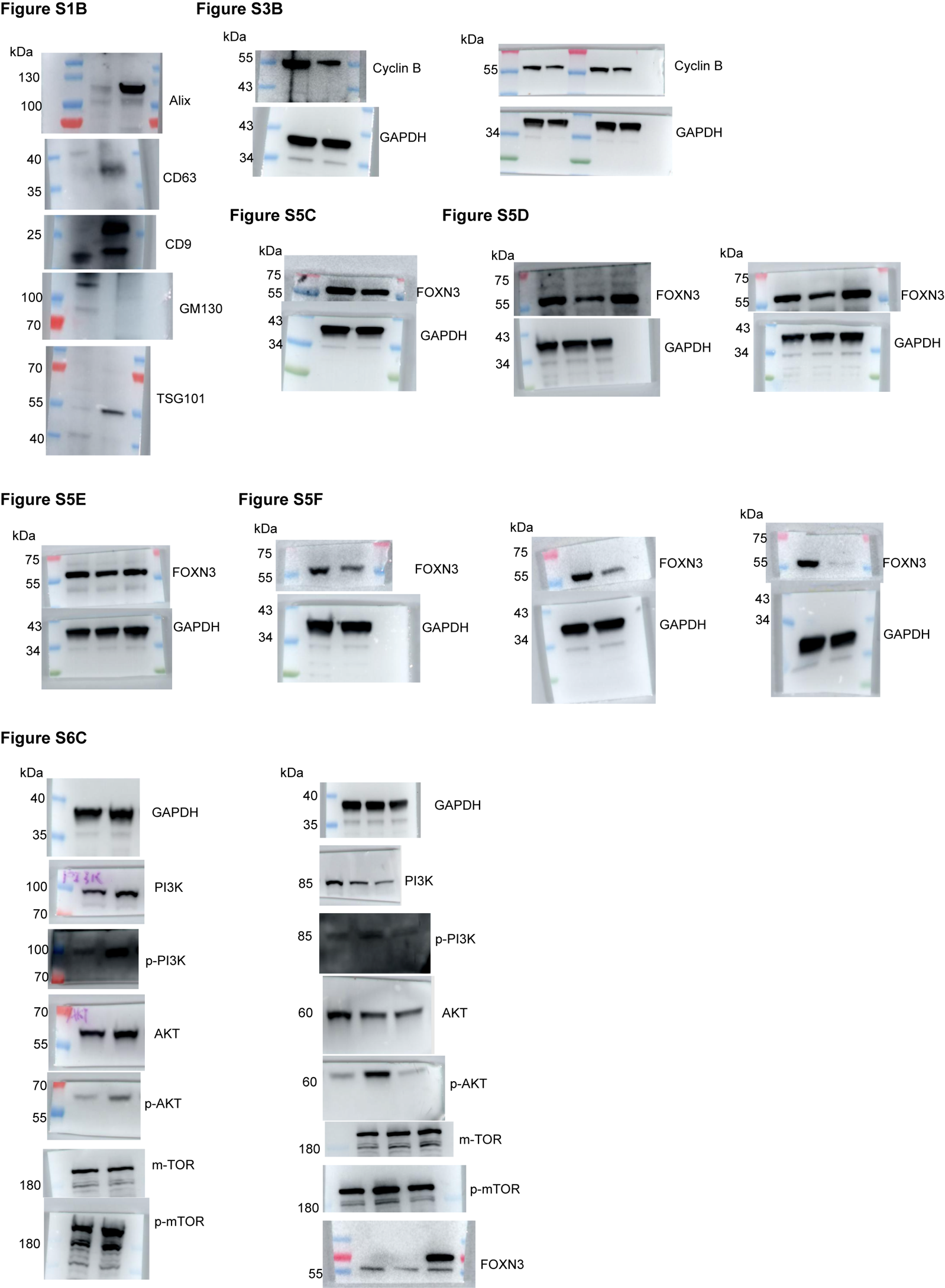

Supplement: Supplementary file 2 — Original Data [file 41419_2026_8597_MOESM2_ESM.docx]
